# From Classical to Alternative Pathways of 2-Arachidonoylglycerol Synthesis: AlterAGs at the Crossroad of Endocannabinoid and Lysophospholipid Signaling

**DOI:** 10.3390/molecules29153694

**Published:** 2024-08-04

**Authors:** Fabienne Briand-Mésange, Isabelle Gennero, Juliette Salles, Stéphanie Trudel, Lionel Dahan, Jérôme Ausseil, Bernard Payrastre, Jean-Pierre Salles, Hugues Chap

**Affiliations:** 1 Infinity-Toulouse Institute for Infectious and Inflammatory Diseases, University of Toulouse, INSERM, CNRS, Paul Sabatier University, 31059 Toulouse, France; fabienne.briand-mesange@inserm.fr (F.B.-M.); isabelle.gennero@inserm.fr (I.G.); juliette.salles@hotmail.fr (J.S.); stephanie.trudel-ausseil@inserm.fr (S.T.); jerome.ausseil@inserm.fr (J.A.); salles.jp@chu-toulouse.fr (J.-P.S.); 2 Centre Hospitalier Universitaire de Toulouse, Service de Biochimie, Institut Fédératif de Biologie, 31059 Toulouse, France; 3 Centre Hospitalier Universitaire de Toulouse, Service de Psychiatrie D’urgences, de Crise et de Liaison, Institut des Handicaps Neurologiques, Psychiatriques et Sensoriels, 31059 Toulouse, France; 4 Centre de Recherches sur la Cognition Animale (CRCA), Centre de Biologie Intégrative (CBI), Université de Toulouse, CNRS, UPS, 31062 Toulouse, France; lionel.dahan@univ-tlse3.fr; 5 I2MC-Institute of Metabolic and Cardiovascular Diseases, INSERM UMR1297 and University of Toulouse III, 31400 Toulouse, France; bernard.payrastre@inserm.fr; 6 Centre Hospitalier Universitaire de Toulouse, Laboratoire d’Hématologie, 31400 Toulouse, France; 7 Centre Hospitalier Universitaire de Toulouse, Unité d’Endocrinologie et Maladies Osseuses, Hôpital des Enfants, 31059 Toulouse, France; 8 Académie des Sciences, Inscriptions et Belles Lettres de Toulouse, Hôtel d’Assézat, 31000 Toulouse, France

**Keywords:** endocannabinoids, 2-arachidonoylglycerol, lysophosphatidylinositol, lysophosphatidic acid, lysophosphatidylcholine, lysophosphatidylserine, GDE3, lipid phosphate phosphatases, ENPP, autotaxin, plasticity-related-gene 1

## Abstract

2-arachidonoylglycerol (2-AG) is the most abundant endocannabinoid (EC), acting as a full agonist at both CB1 and CB2 cannabinoid receptors. It is synthesized on demand in postsynaptic membranes through the sequential action of phosphoinositide-specific phospholipase Cβ1 (PLCβ1) and diacylglycerol lipase α (DAGLα), contributing to retrograde signaling upon interaction with presynaptic CB1. However, 2-AG production might also involve various combinations of PLC and DAGL isoforms, as well as additional intracellular pathways implying other enzymes and substrates. Three other alternative pathways of 2-AG synthesis rest on the extracellular cleavage of 2-arachidonoyl-lysophospholipids by three different hydrolases: glycerophosphodiesterase 3 (GDE3), lipid phosphate phosphatases (LPPs), and two members of ecto-nucleotide pyrophosphatase/phosphodiesterases (ENPP6–7). We propose the names of AlterAG-1, -2, and -3 for three pathways sharing an ectocellular localization, allowing them to convert extracellular lysophospholipid mediators into 2-AG, thus inducing typical signaling switches between various G-protein-coupled receptors (GPCRs). This implies the critical importance of the regioisomerism of both lysophospholipid (LPLs) and 2-AG, which is the object of deep analysis within this review. The precise functional roles of AlterAGs are still poorly understood and will require gene invalidation approaches, knowing that both 2-AG and its related lysophospholipids are involved in numerous aspects of physiology and pathology, including cancer, inflammation, immune defenses, obesity, bone development, neurodegeneration, or psychiatric disorders.

## 1. Introduction

The endocannabinoid (EC) system, which was discovered from the psychotropic effects of Δ-9-tetrahydocannabinol (THC, the main psychoactive compound of cannabis sativa), includes two G-protein-coupled receptors (GPCRs), called CB1 and CB2, numerous lipid mediators called ECs, mainly anandamide and 2-arachidonoylglycerol (2-AG), as well as various enzymes involved in the metabolism of ECs [1]. Beside their well-established role in the central nervous system via the CB1 receptor, ECs are also active in the immune system, where CB2 is the predominant receptor [2,3]. However, the situation is not so clear-cut, since CB1 is also present in peripheral organs such as the liver, intestine, and adipose tissue, where it regulates energetic metabolism, whereas CB2 is also detected in the central nervous system, where it could be involved in immune defense and in neuroinflammation [1,2]. Recent advances revealed a much more complex situation with additional receptors, such as various GPCRs (GPR55, GPR18, GPR119), transient receptor potential cation channel subfamily V (TRPV1, TRPV4), peroxisome proliferator-activated receptors (PPARα, PPARγ), as well as an increasing number of anandamide and 2-AG congeners, forming the endocannabinoidome, with all of them contributing to a recently recognized expanded EC system [4,5,6]. In addition, the lipidic nature of ECs requires renewed attention to proteins involved in EC intracellular and extracellular transport [7,8,9,10].

2-AG is 170-fold more abundant than anandamide in the brain [11], in agreement with other studies [12], and was thus recognized as the main player involved in the retrograde inhibition of neurotransmitter release upon interaction with CB1, present in both the excitatory and inhibitory presynaptic terminals [4,5,6,7,8,9,10,11,12,13,14,15,16,17,18,19]. Although this central mechanism probably forms the basis of 2-AG involvement in memory, pain, anxiety, mood, stress, regulation of hyperexcitability, neuroprotection, or addiction, its peripheral interactions with both CB1 and CB2 receptors also revealed its role in regulating the energetic metabolism of the liver, muscle, or adipose tissue, intestinal function, cell proliferation, immune defenses, bone development, or inflammation. So, defining pharmacological targets which are able to modify 2-AG interaction with its receptors or 2-AG metabolism might bring renewed interest in the field of various pathologies, including psychiatric disorders, neurodegenerative diseases, various forms of pain, obesity, inflammatory bowel diseases, or cancer [1,2,3,4,5,6,10,12,13,14,15,16].

**Figure 1:**
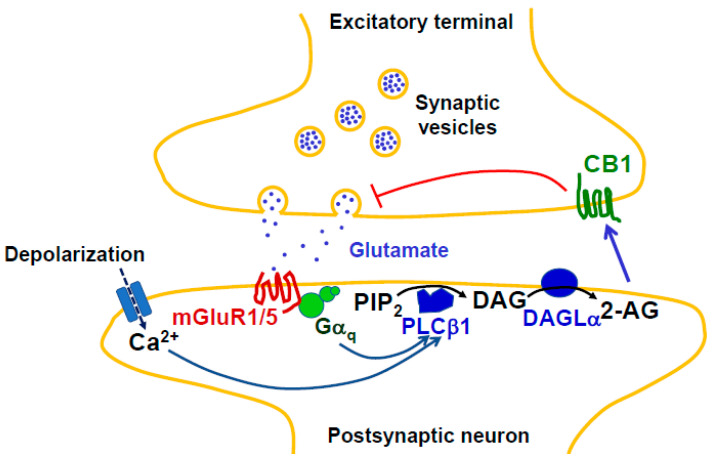
Main enzymes of the canonical pathway of 2-AG synthesis involved in synaptic retrograde signaling. Glutamate released from excitatory terminal interacts with its ionotropic receptors AMPAR and NMDAR (not represent here), which results in the depolarization of postsynaptic neurons, allowing Ca^2+^ influx through NMDAR and voltage-gated Ca^2+^ channels. Simultaneous binding of glutamate (blue points) to mGluR1/5 promotes the Gα_q_-dependent activation of PLCβ1. The latter enzyme activity is increased by Ca^2+^ but requires Gα_q_ interaction to hydrolyze PIP_2_, thus acting as a coincidence detector [15,25]. 1-Stearoyl-2-arachidonoyl-*sn*-glycerol, the major DAG molecular species generated from phosphatidylinositol 4,5-bisphosphate (PIP_2_), can then be converted into 2-AG by diacylglycerol lipase α (DAGLα). The efficiency of this enzymatic cascade rests on the proper positioning of the various actors involving, among other mechanisms, interaction between DAGLα and mGluR1/5 via the scaffold Homer proteins [26,27,28,29]. 2-AG then diffuses through the synaptic cleft to presynaptic CB1, thus inhibiting the further release of glutamate. Mechanisms of CB1-induced presynaptic changes controlling short- and long-term synaptic plasticity are described in detail elsewhere [19]. Not shown here, CB1 is also present in GABAergic inhibitory terminals, resulting in the suppression of inhibition [30]. Abbreviations: 2-AG, 2-arachidonoylglycerol; AMPAR, α-amino-3-hydroxy-5-methyl-4-isoxazolepropionic acid receptor; CB1, cannabinoid receptor 1; DAG, diacylglycerol; DAGLα, DAG lipase α; GABA, γ-amino butyric acid; Gα_q_, α_q_ subunit of heterotrimeric G protein; mGluR1/5, metabotropic glutamate receptor 1 or 5; NMDAR, *N*-methyl-D-aspartate receptor; PIP_2_, phosphatidylinositol 4,5-bisphosphate; PLCβ1, phospholipase Cβ1.

Figure 1 depicts the paradigm of the enzymatic cascade of 2-AG synthesis occurring during synaptic retrograde signaling involving metabotropic glutamate receptors. This involves the production of the diacylglycerol (DAG) 1-stearoyl-2-arachidonoyl-sn-glycerol from phosphatidylinositol 4,5-bisphosphate (PIP_2_) by a phospholipase Cβ1 (PLCβ1), followed by the hydrolysis of DAG by DAG lipase α (DAGLα). The efficiency of this pathway in 2-AG production is greatly favorized by the exceptional abundance of arachidonic acid in phosphoinositides, where 1-stearoyl-2-arachidonoyl species represent between 70 and 90% of total molecular species ([20,21] and references herein). Such a model received a very elegant confirmation through the use of a 2-AG sensor, allowing to follow the spatiotemporal imaging of synaptic retrograde signaling [22,23]. As another elegant approach, mass spectrometry imaging was recently applied to detect increases in 2-AG in various brain regions in response to chronic restraint stress [24].

If the picture is valid in a great majority of cases, the careful inspection of the available literature indicates that a much greater diversity might exist at the level of PLC and DAGL (illustrated in Table 1). This will be the first point developed in the present review (Section 2).

**Figure 2:**
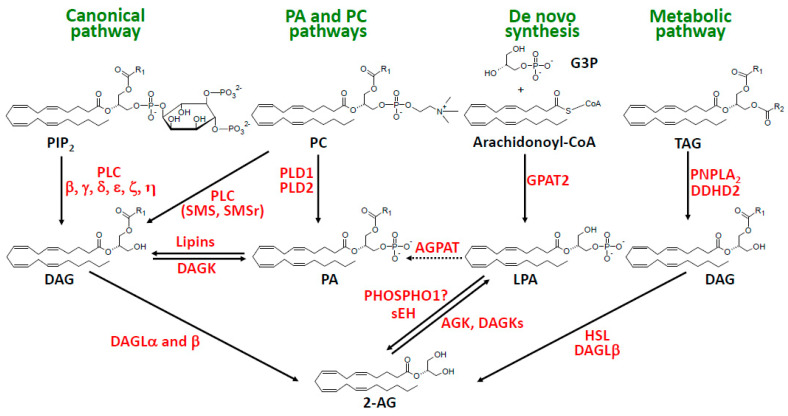
Canonical and other intracellular pathways of 2-AG synthesis. The acylation of LPA to PA by AGPAT is indicated with dotted lines to describe either the pathological conditions (AGPAT2 inactivating mutations responsible for congenital lipodystrophy [33]) or, for some yet unknown reasons, physiological situation limiting de novo synthesis to the production of LPA and 2-AG. Abbreviations: 2-AG, 2-arachidonoylglycerol; AGK, acylglycerol kinase; AGPAT, 1-acylglycerol-3-phosphate acyltransferase; DAG, diacylglycerol; DAGK, DAG kinase; DAGL, DAG lipase; DDHD, DDHD containing; G3P, sn-glycerol-3-phosphate; GPAT, sn-glycerol-3-phosphate acyltransferase; HSL, hormone-sensitive lipase; LPA, lysophosphatidic acid; PA, phosphatidic acid; PC, phosphatidylcholine; PHOSPHO1, phosphocholine and phosphoethanolamine phosphatase; PIP_2_, phosphatidylinositol 4,5-bisphosphate; PLC, phospholipase C; PLD, phospholipase D; PNPLA_2_, patatin-like PLA_2_; sHE, soluble epoxide hydrolase; SMS, sphingomyelin synthases (SMS1 and SMS2); SMSr, SMS related protein; TAG, triacylglycerol.

As shown in Figure 2 and described in Section 3, other sources of DAG hydrolyzed by DAGL could be PA dephosphorylated by lipins or PC cleaved by SMS (sphingomyelin synthase) or SMSr (SMS-related protein PLC) acting as PLC. In a recent review, Baggelaar et al. [13] proposed an additional pathway (the ‘metabolic pathway’) where intermediary DAGs are generated from triacylglycerols through the action of various lipases (depicted in Figure 2). This will be discussed in light of recently accumulated knowledge concerning those various lipases (Section 4). Finally, a recent study reported unexpected data giving credit to a possible contribution of glycerolipid de novo synthesis to 2-AG biosynthesis (Figure 2), which will be discussed in Section 5.

**Figure 3:**
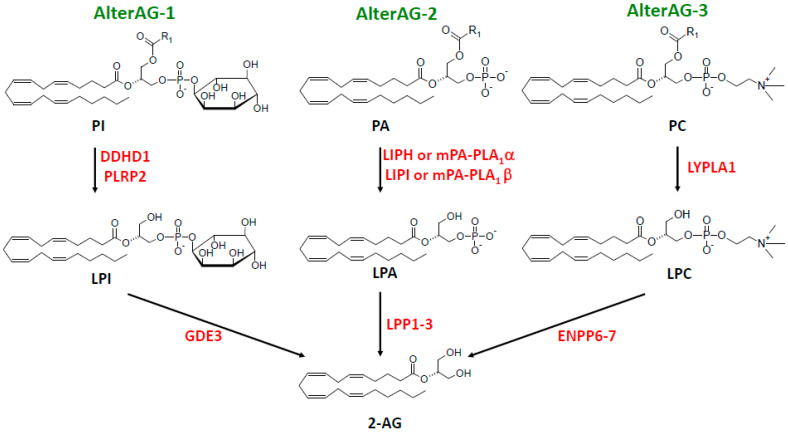
The three extracellular AlterAG pathways. Abbreviations: 2-AG, 2-arachidonoylglycerol; AlterAG, alternative pathway of 2-AG synthesis; DDHD, DDHD containing; ENPP6–7, ecto-nucleotide pyrophosphatase/phosphodiesterases 6 and 7; GDE3, glycerophosphodiesterase 3; LIPH and LIPI, lipases H and I; LPA, lysophosphatidic acid; LPC, lysophosphatidylcholine; LPI, lysophosphatidylinositol; LPP1–3, lipid phosphate phosphatases 1–3; LYPLA1, lysophospholipase A1; mPA-PLA_1_, membrane-associated PA-selective PLA1 (α or β); PA, phosphatidic acid; PC, phosphatidylcholine; PI, phosphatidylinositol; PLRP2, pancreatic lipase related protein 2.

In addition, we and another group recently identified GDE3 as a main actor in an alternative cascade leading to 2-AG production and linked to LPI metabolism and signaling [31,32]. But, at least two other alternative pathways (AlterAGs) linking 2-AG and LPL mediators must be considered. As shown in Figure 3, we propose the names AlterAG-1, -2, and -3 for those pathways whose last step occurs in the extracellular space. These pathways draw particular attention to the importance of the positional isomerism of 2-AG and related LPLs, implying problems of chemical stability as well as enzyme and receptor specificity. This point will be discussed in Section 6 before the description of AlterAG pathways in Section 7.

**Table 1:** Various enzymes involved in the PLC/DAGL pathway of 2-AG production.

Enzymes		Cells or Tissues	Subcellular Localization Following Activation	Conditions of Activation	(Patho)physiological Involvement	Ref
PLC	PLCβ1	Hippocampal neurons	Plasma membrane	G_q/11_-coupled receptors (mGluR1/mGluR5 or M1/M3) *plus* depolarization	Complex picture describing KO mice and human pathologies reviewed in detail by Katan and Cockroft [34]	[25]
	PLCβ4	Cerebellum (Purkinje cells)	Plasma membrane	G_q/11_-coupled receptor (mGluR1) *plus* depolarization		[35]
	PLCδ	Cultured hippocampal neurons	Plasma membrane	Depolarization (DSI)No effect of δ1, δ3, δ4 KO		[36]
	PLCε	Ventral tegmental area (VTA) dopamine neurons	Plasma membrane	Depolarization (DSI) facilitated by cAMP-Epac2-Rap-PLCε cascade	Contribution to cocaine-induced disinhibition of VTA dopamine neurons	[37]
	PLCγ1	Hippocampus (mossy fiber synapses onto stratum lucidum interneurons Calyx of Held (giant glutamatergic synapse)	Plasma membrane	High-frequency stimulation leading to long-term depression via endogenous BDNF release BDNF application during depolarization		[38] [39]
	PLCγ2	Macrophages, microglia	Plasma membrane	FcγR cross-linking generating a DAG–MAG–eicosanoid network	Hyperactive variants in autoimmune and inflammatory diseases or protecting from Alzheimer disease	[40]
**DAGL**	DAGLα	Hippocampus, cerebellum, striatum slices or cultured neurons Striatonigral direct-projecting pathway medium spiny neurons	Plasma membraneRapid turnover upon membrane trafficking	G_q/11_-coupled receptors or depolarization Depolarization	Production of 2-AG and AAAxon growth/guidance, neurogenesisAnxiety, fear, extinction, impairmentMetabolic phenotype similar to CB1-KO miceNeuro-ocular DAGLA related syndrome Ethanol effects	[41,42,43,44] [45,46,47,48] [49,50] [51] [52] [53]
	DAGLα	Astrocytes Tanycytes	Plasma membrane Plasma membrane		Affective disorders, hedonic feeding Inhibition TRH release	[54,55] [56]
	DAGLβ	Brain, liver, macrophages, microglia, *S. nigra* dopaminergic neuronsCargo protein of AP-4 vesicles	Plasma membrane AP-4 vesicles during axonal anterograde transport		Altered neurogenesis2-AG, AA, and eicosanoid production Parkinson disease2-AG-dependent axon growth (altered in AP4-deficiency)	[42] [57,58] [59] [60]
	ABHD6	Neuro-2a cells	ND	Retinoic acid-induced differentiation		[61]
	ABHD11	Ubiquitous expression	Mitochondria		No change in tissue 2-AGKO mice resistant to obesity	[62]
	DDHD2	Brain	Cytosol	In vitro determination	DAGL in vitro, TAGL in vivoPlastic paraplegia	[63,64,65,66]
	HSL	Neurons and astrocytes	Pre- and post-synaptic membranes		Short- and long-term memory in aged mice	[67,68]

Abbreviations: 2-AG, 2-arachidonoylglycerol; AA, arachidonic acid; ABHD, α/β-Hydrolase Domain-Containing; AP4, adaptator protein complex 4; BDNF, Brain-Derived Neurotrophic Factor; DAG, diacylglycerol; DAGL, diacylglycerol lipase; DDHD, DDHD containing; DSI, depolarization-induced suppression of inhibition; Epac, exchange protein directly activated by cAMP; HSL, hormone-sensitive lipase; *S. nigra*, *substantia nigra*; M1/M3, muscarinic receptors (1 or 3); MAG, monoacylglycerol; mGluR1/5, metabotropic glutamate receptor (1 or 5); PLC, phospholipase C; TAGL, triacylglycerol lipase.

## 2. Variations in the Use of PLC and DAGL Isoforms Involved in 2-AG Synthesis

### 2.1. Phosphoinositide-Specific PLCs

There are 16 different members of PLC distributed between six classical families (β,γ,δ,ε,ζ,η) and one atypical family [34]. In their very complete review, Kano et al. [15] recalled the various distributions of the four PLCβ isoforms, which are not overlapping in central nervous system (CNS). As shown in Table 1, PLCβ1 in hippocampal neurons [25] and PLCβ4 in Purkinje cells [35] appear to follow the same regulation downstream of Gα_q_-coupled metabotropic receptors. This is the case depicted in Figure 1, which is further confirmed by the forebrain-specific inactivation of Gα_q_-/Gα_11_ family G proteins [69].

Retrograde signaling by 2-AG was discovered using experimental models of depolarization-induced suppression of excitation (DSE) or inhibition (DSI) [30]. In that case, μM cytosolic calcium concentrations ([Ca^2+^]_i_) are reached through opening voltage-gated Ca^2+^ channels, but the PLC at the source of the DAGL substrate has not been identified. In contrast to PLCβs, PLCδ1 is directly activated by μM [Ca^2+^]_i_ [34] and thus appeared as a good candidate to achieve this goal. However, hippocampal DSI was unal-tered in PLCδ1-, δ3- and δ4-knockout mice (Table 1 and [36]). As discussed by Hashimotodani et al. [36], double- or triple-PLCδ-knockout mice were not tested (the double mutant δ1−δ3 is lethal [34]), leaving open the question of whether PLC is involved in 2-AG-dependent DSE or DSI.

Among other members, PLC ζ and PLCη also display a high sensitivity to Ca^2+^. Whereas PLC ζ is sperm-specific, PLCη1 and PLCη2 are present in the brain, especially the latter one, which is developmentally regulated and detected in the hippocampus, cerebral cortex, olfactory bulb, habenula, retina, pituitary, and neuroendocrine cells [34]. In vitro, optimal [Ca^2+^] are 1 μM and 10 μM for PLCη2 and PLCδ1, respectively [70]. In intact cells activated by various GPCR ligands, PLCη1 is stimulated by Ca^2+^ mobilized from internal stores [71], whereas PLCδ1 activity depends on external Ca^2+^ influx [72,73]. It is tempting to discuss those properties in light of the study showing that retrograde synaptic signaling can be equally achieved either under current artificial conditions elevating postsynaptic [Ca^2+^]_i_ over 5 μM until almost 50 μM (single pulses of 100 to 2000 ms, respectively) or upon sustained but limited (around 1 μM) [Ca^2+^]_i_ elevation obtained by a series of brief depolarizations [74]. A possible role of PLCη1 and PLCη2 in DSE/DSI explored under both conditions mentioned above and using corresponding knockout mice would thus deserve attention.

Another PLC (PLCε) displays a unique mechanism of activation involving exchange protein directly activated by cAMP (Epac), a direct effector of cAMP [34]. A cascade involving cAMP-Epac2-PLCε-2-AG was shown to occur in dopamine neurons of the ventral tegmental area, where it facilitates DSI and long-term depression at inhibitory synapses (I-LTD) [37]). As outlined in Table 1, this cascade appears to participate in the cocaine-induced disinhibition of VTA dopamine neurons.

PLCs from the γ family are activated downstream of receptor or non-receptor protein tyrosine kinases in a mechanism involving their two src-homology-2 (SH2) domains. Two studies mentioned in Table 1 reported convincing evidence that synaptic retrograde signaling might involve Brain-Derived Neurotrophic Factor (BDNF) binding to its postsynaptic TrkB receptor, thus promoting the activation of PLCγ1 followed by the DAGL generation of 2-AG [38,39].

One can thus conclude that β, γ, and ε PLCs must be considered as actors of the PLC-DAGL cascade contributing to synaptic plasticity. Curiously, DSI and DSE, which allowed the discovery of CB1-dependent synaptic retrograde signaling, did not yet reveal the identity of involved PLC, although PLC ζ and PLCη should undergo further investigations.

Finally, a recent study was focused on PLCγ2, which is mainly expressed in hematopoietic cells at the periphery and specifically in microglia within CNS. Using exogenous expression in transfected cells, as well as macrophages and microglia, Jing et al. [40] provided a very extensive description of endocannabinoid and eicosanoid networks resulting from a triple enzyme cascade, i.e., PLCγ2-DAGL-MAGL (monoacylglycerol lipase). One striking observation was the balance occurring between that pathway of eicosanoid production and the one involving PLA2G4A, as revealed upon the disruption of the PLCγ2 gene. As recalled in a recent review [75], our group was the first to propose DAGL as another pathway of arachidonic acid liberation [76,77], an idea which was then put forward mainly by the Majerus group [78,79]. Those proposals were made almost 45 years ago at a period where EC was not yet discovered. The favorite cell model used in those previous studies was blood platelets, which revealed how PI 3-kinase modulates the activity of PLCγ2 under conditions of FcγRIIA engagement [80,81], similar to those depicted by Cravatt’s group with macrophages and microglia [40]. Among other interesting observations of the latter-mentioned study, several pathological variants of PLCγ2 were found to display gain of function, which will be interesting to keep in mind in understanding the pathophysiology of some autoimmune and inflammatory diseases, as well as Alzheimer disease, as recalled in Table 1. Thus, 2-AG production is not the only result of PLC-DAGL cascade, which can also display strong interactions with eicosanoids [82].

For the sake of clarity, the main characteristics of PLC isoenzymes discussed above are summarized in Table 2.

As discussed below, the nature of DAGL involved in 2-AG synthesis deserves particular attention.

### 2.2. Duality between DAGLα and DAGLβ

As recalled in more detail in [13] and summarized in Table 1, two DAGL isoforms (α and β) are the products of different genes [41], whose invalidation assigned a central role to DAGLα in synaptic retrograde signaling described above [42,43]. This was fully confirmed with the most specific irreversible inhibitors available so far [44]. On a functional point of view, the genetic disruption of DAGLα gene, which was accompanied by a drastic fall of brain 2-AG content, was found to reduce neurogenesis [42] and to reproduce alterations of CB1 receptor signaling on anxiety [49,50], energetic metabolism, and food intake [51]. As reviewed by Oudin et al. [45], DAGLα also contributes to the regulation of axon growth and guidance during development and to adult neurogenesis (Table 1). The very recent identification of DAGLα mutations responsible for a neuro-ocular DAGLA-related syndrome (NODRS) is a good example of a genetic disease linked to the EC system [52]. Interestingly, mutations identified in NODRS patients affected the C-terminal part of the protein, leaving intact its catalytic activity but altering its subcellular localization, which became perinuclear (instead of membrane-bound for the wild-type protein), at least in a transfected model of HEK293T cells. This puts the accent on the importance of DAGLα localization on the postsynaptic membrane, probably under the control of Homer proteins, as recalled in the legend of Figure 1 [26,27,28,29]. Whether this is related to the possible existence of different DAG pools involved in mGluR1-dependent retrograde signaling or DSI, respectively, still remains an open question [83].

Besides the well-established role of DAGLα in neurons, a low level of its gene expression was detected in a subpopulation of astrocytes [54], in agreement with the fact that DAGLα is the isoform producing 2-AG in isolated astrocytes [84]. Conditional knockout did not alter brain 2-AG content, but, as recalled in Table 1, this had profound behavioral consequences such as depressive-like behavior, alterations in maternal care behavior, and hedonic feeding [54,55]. This very interesting observation is thus to add to the possible involvement of ECs in the interplay between astrocytes and neurons [58]. In another recent study, DAGLα present in tanycytes was found to regulate the hypothalamic–pituitary–thyroid axis, as recalled in Table 1 [56].

DAGLβ is also expressed in the brain, where it predominates in microglia [84], but also displays more peripheral localizations such as the liver [41] or peritoneal macrophages [57]. Since the genetic or pharmacological inhibition of DAGLβ also alters the liberation of arachidonic acid and its conversion into eicosanoids, this might indicate a main role of this isoform in the regulation of inflammation. However, one cannot adopt such a simple view when observing that DAGLα also exerts a duplicate function in the interconnected EC and eicosanoid metabolism [44,57]. The link between the two families of lipid mediators is provided by 2-AG hydrolysis, which allows the simultaneous regulation of the 2-AG level and the liberation of arachidonic acid. This aspect is out of the scope of this review, but, just to give an idea of the complexity of the various enzyme combinations, the action of microglia DAGLβ seems to be coupled with intracellular MAGL for the production of eicosanoids and with extracellular ABHD12 for the regulation of secreted 2-AG [84].

As recalled in Table 1, DAGLβ, which is the predominant DAGL in human and mouse substantia nigra dopaminergic neurons, displays loss of function mutations responsible for early onset Parkinsonism, opening an interesting field in our comprehension of Parkinson disease pathophysiology [59]. The same enzyme was also identified as a cargo of AP-4 vesicles and revealed a direct link between 2-AG production and a severe neurodevelopmental and neurodegenerative disorder occurring in AP-4-deficient patients [60]. The two latter studies bring substantial advances in the field of EC systems by combining specific cellular or subcellular expression data to the identification of genetic diseases involving DAGLβ itself or a protein partner such as AP-4.

### 2.3. A Nuclear PLCβ-DAGLα Cascade

In strong contrast with the need of PLC-DAGLα localization in the postsynaptic membrane mentioned above, three isoforms of PLC (β1, β2, and β4) were detected together with DAGLα in very specific domains of the nuclear matrix from rat cortical neurons [85,86], in line with our previous studies on the nuclear phosphoinositide cycle [87]. The authors provided in vitro evidence that efficient coupling between the two enzymes allowed production of 2-AG and suggested two possible functional consequences as follows: either direct interaction of 2-AG with transcription factor PPARγ or release of AA followed by conversion into PGJ2, another ligand of PPARγ. Although this very interesting view awaits further study, it adds to the large spatial and functional diversity of the PLC-DAGL pathway described in the present review.

### 2.4. Other Lipases Possibly Involved in 2-AG Synthesis

Four other enzymes have been suggested to possibly achieve DAG conversion to 2-AG: ABHD6 was first identified as a MAGL but was found to also display DAGL activity; ABHD11 and DDHD2 were purified on the basis of their in vitro activity; finally, hormone-sensitive lipase (HSL) is well known as a main enzyme involved in lipolysis occurring in adipose tissue. They are discussed in more detail below.

#### 2.4.1. ABHD6

In a model of the retinoic acid-induced differentiation of the murine neuroblastoma cell line Neuro-2a, ABHD6 was found to display typical DAGL activity and to contribute, probably in conjunction with DAGLβ, to retinoic acid-induced 2-AG accumulation [61]. This was a quite unexpected finding since ABHD6 was initially discovered as a MAGL contributing to the regulation of the 2-AG level [17,88]. However, besides this dual MAGL/DAGL character, ABHD6 is also active against other lipid substrates such as lysophospholipids or bis(monoacylglycero)phosphate (BMP), also called lysobisphosphatidic acid (LBPA) [17,88]. So, further studies are still needed to understand the precise role of ABHD6 in 2-AG metabolism, which might vary with cell localization.

#### 2.4.2. ABHD11

**Figure 4:**
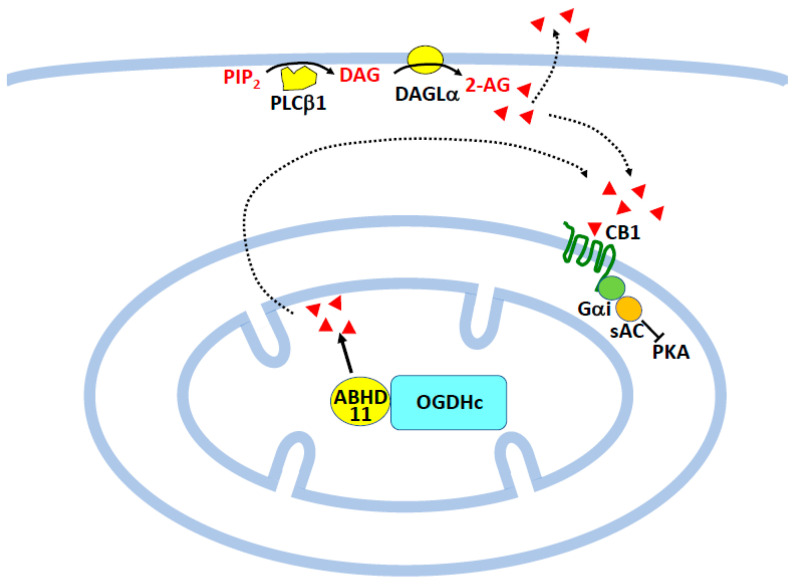
Possible relationship between ABHD11 and mitochondrial CB1. Besides the canonical pathway present in the plasma membrane (see also Figure 1), ABHD11 might be another source of 2-AG for mitochondrial CB1 receptors. 2-AG (red triangles) from both origins is postulated to interact with mitochondrial CB1, resulting in the sequential inhibition of sAC and PKA. The scheme is inspired from previous representations found in the literature [97,98]. As commented in the text, ABHD11 in mitochondrial matrix is associated with OGDHc. Abbreviations: 2-AG, 2-arachidonoylglycerol; ABHD11, ABHD, α/β-Hydrolase Domain-Containing; CB1, cannabinoid receptor 1; DAG, diacylglycerol; DAGL, DAG lipase; Gα_i_, α_i_ subunit of heterotrimeric G protein; OGDHc, oxoglutarate dehydrogenase complex; PIP_2_, phosphatidylinositol 4,5-bisphosphate; PKA, protein kinase A; PLC, phospholipase C; sAC, soluble adenylate cyclase.

Another hydrolase of the same family, ABHD11, was purified as a DAGL by the Sanofi Research group [62]. Despite it rather high in vitro activity, mice bearing an invalidated ABHD11 gene did not display any change in 2-AG level in various organs including the brain, liver, muscle, or adipose tissue. This might simply reflect the fact that ABHD11 acts on a minor pool of DAG, similar to astrocyte DAGLα discussed above [54]. Interestingly, the mutant mice were revealed to be resistant to diet-induced obesity, albeit with no evidence of change in EC tone. Apparently, the mechanism might involve an alteration of bile acid synthesis, resulting in a reduced intestinal absorption of dietary lipids [62]. ABHD11 is localized in the mitochondrial matrix [62], where its specific interaction with OGDHc (oxoglutarate dehydrogenase complex) regulates glutamine metabolism [89,90]. As illustrated in Figure 4, besides its classical positioning at the plasma membrane, CB1 has also been detected in mitochondria from brain cells, muscle, sperm, oocytes, and adipocytes, where signaling involves heterotrimeric Gαi coupled to soluble adenylate cyclase (sAC) and protein kinase A (PKA) (see [91,92] for recent references). Although the DAG substrate of mitochondrial ABHD11 is absolutely unknown, it is thus tempting to emphasize a possible link between mitochondrial ABHD11 and mitochondrial CB1, as depicted in Figure 4 below. However, the role of ABHD11 in an alternative pathway of 2-AG synthesis remains an open question and other, even more complex mechanisms could be emphasized [93].

#### 2.4.3. DDHD2

Similar to ABHD11, DDHD2 was also purified to homogeneity by following its DAGL activity and was logically suggested as a possible candidate involved in 2-AG synthesis [63,64]. Recombinant DDHD2 is mainly active on DAG, but also hydrolyzes TAG and PA as a phospholipase A_1_ (PLA_1_). However, further studies using DDHD2-KO mice and cells transfected with DDHD2 bearing inactivating mutations found in a complex form of hereditary spastic paraplegia (HSP) unambiguously revealed that DDHD2 behaves in vivo as a TAG lipase [65,66]. Interestingly, our group was involved in the study of Sanfilippo syndrome type B, also called mucopolysaccharosidosis type IIIB (MPSIIIB) [94,95]. A recent report describing the simultaneous pathogenic mutations of DDHD2 and NAGLU (the disease-causing gene in MPSIIIB) in a very consanguineous family, thus appears as a rare interesting curiosity [96].

#### 2.4.4. HSL

Finally, a very recent study reported the presence of HSL in various regions of the brain, both in neurons and glial cells, with a slightly higher abundance in postsynaptic membranes [67]. For the record, HSL is a main enzyme of adipose tissue involved in lipolysis. It displays TAG, DAG, and MAG lipase activities, but in vivo function involves its DAGL activity operating between two reactions catalyzed by ATGL (adipose triglyceride lipase) and classical MAGL, allowing the release of glycerol and of the three fatty acids from stored TAG [99,100,101]. Interestingly, although HSL-specific DAGL activity is 20-fold lower in the brain compared to adipose tissue, it represents around two-thirds of total DAGL activity in all brain regions [67]. Among various non-significant differences, only the brain 2-linoleoylglycerol levels were reduced by 20% in the hippocampus of HSL^−/−^ mice, this was limited to 13–14% for both linoleoylglycerol and 2-AG in cortex, but a number of eicosanoids were also modified [67]. The invalidation of the HSL gene was accompanied by the impairment of short-term and long-term memory in old mice, but not in young mice [67,68]. As for ABHD11 and DDHD2, these interesting observations are difficult to link to EC system and 2-AG synthesis, although the lack of lipidomic change can simply reflect the involvement of a minor pool of 2-AG.

To conclude the point concerning other potential lipases, we want to mention a study showing that brain slices incubated at 20 °C for almost 3 h accumulated huge amounts of 2-AG, which were detected only in the presence of MAFP (methylarachidonoylfluorophosphonate), a ‘broadly acting serine hydrolase inhibitor’ previously described as ‘a global inhibitor of 2-AG hydrolysis’ [102]. Most remarkably, this was accompanied by the activation of CB1, as shown by [^35^S]GTPγS autoradiography. 2-AG production was unchanged in DAGLα-KO and DAGLβ-KO mice and was almost abolished by tetrahydrolipstatin (THL), an irreversible inhibitor of the two lipases [41]. This suggested that other THL-sensitive lipases were involved in 2-AG synthesis occurring under these conditions. Table 3 recapitulates the literature data obtained mainly through activity-based protein profiling (ABPP) with 35 different serine hydrolases [103].

In addition to DAGLα and β, DDHD2 thus appears as the only THL-sensitive lipase acting on DAG and (or) TAG, with in vitro inhibition with DAG as a substrate occurring at an IC_50_ of 7.8–10 nM THL [63]. Based on these data, HSL is probably not involved in 2-AG accumulation occurring in brain slices; however, demonstrations showing that DDHD2 is the only lipase candidate still requires demonstration.

As discussed by Aaltonen et al. [102], the massive production of 2-AG occurring in incubated brain slices probably corresponds to what is observed following decapitation, reflecting biochemical events occurring upon death [104,105]. Although far from physiological conditions, cellular and biochemical mechanisms behind those changes would deserve particular attention, since they might occur under pathological situations such as ischemia or trauma. Among the hypothetical mechanisms possibly involved in massive 2-AG production, AlterAGs could be emphasized, as discussed further and as already suggested [102]. It would also be important to identify from which lipid pool 2-AG originates: phosphoinositides via the action of PLCs preceding that of lipases, PC converted into DAG through reversible reaction of phosphocholine transferase [106], or even TAG hydrolyzed by DDHD2, as discussed earlier. In this context, a very careful study conducted on gerbil cerebral cortex during ischemia indicated that DAG issued from phosphoinositide hydrolysis was the main source of stearic acid, whose liberation preceded that of arachidonic acid [107]. Remarkably, that study was performed before the discovery of the EC system and would justify novel investigations using modern possibilities offered by lipidomic analysis. One major interest would be to reveal biochemical mechanisms resulting in massive brain accumulation of 2-AG, with two possible consequences: i) modulation of ischemia–reperfusion damages by CB1; ii) consciousness changes preceding death as suggested by near-death experiences [108].

To conclude this section, several lipases other than DAGLα and β can be emphasized in some pathways leading to 2-AG synthesis. Their possible role in putative metabolic pathways will be discussed further in Section 4. However, another possible origin of the DAGL substrate might involve PA generated by the PLD hydrolysis of PC and is described below.

## 3. The PA and PC Pathways

Using the mouse neuroblastoma cell line N18TG2 stimulated with the calcium ionophore ionomycin, Bisogno et al. [109] provided evidence that the DAGL substrate was derived from a pool of PA dephosphorylated by PA phosphatase, as deduced from the inhibitory action of propranolol and N-ethyl-maleimide. As shown in Figure 2, this reaction would be catalyzed by one isoform of lipins, the novel name of PA phosphatases [110,111,112,113]. However, the origin of PA could not be clearly identified. A very similar conclusion was reached with the rat microglial cell line (RTMGL1), where 2-AG synthesis was unaltered by PLC inhibitor U73122 but was strongly reduced by propranolol [114]. However, the first evidence for a functional pathway involving the successive actions of a PC-specific PLD (likely PLD2 in this case), lipin, and DAGL (see Figure 2) was provided by Zhang et al. [115] in rat thalamic paraventricular nucleus neurons displaying unique electrophysiological properties modulated by intracellular CB1, and probably CB2, receptors.

As also shown in Figure 2, the generation of DAG upon the hydrolysis of PC (or other glycerophospholipids) by a putative PLC is currently suggested from the use of a specific inhibitor of Bacillus cereus PLC, tricyclodecan-9-yl-xanthogenate (D609) [116]. D609 was found to be ineffective in brain slices, where PIP_2_-specific PLC is involved [11], but inhibited 2-AG production in a model of local mouse ear inflammation induced by the topical application of 12-O-tetradecanoylphorbol-13-acetate (TPA) [117]. As indicated in Figure 2, this reaction, which might concern not only PC but other glycerophospholipids such as PE, could be catalyzed by one of the three SPS, which have been recently recognized for their PLC activity [118,119,120,121,122]. However, TPA-induced 2-AG synthesis upon ear inflammation also involved PIP_2_-specific PLC and PLD–lipin pathways, as shown by the use of various pharmacologic inhibitors, in a model leading to the production of several other MAG species [117].

Using mouse microglial cells in primary culture and a DAGK inhibitor, Witting et al. [123] observed that DAGK shunted DAG from the DAGL pathway, thus revealing a possible regulation mechanism of the DAG level. Whether this is related to the reported interaction of SMS with DAGK might thus deserve to be questioned [124,125].

In conclusion of this section, the various reactions involving PC and (or) PA have been suggested mainly from studies based on the use of pharmacological inhibitors, whose specificity might not be absolute, at a time when the corresponding enzymes (PLDs, lipins, DAGKs, SMS) were not identified at the molecular level. It is thus difficult to draw very strong conclusions in the absence of data concerning their expression in the investigated models and of experiments based on the specific knockdown or knockout of the corresponding genes. These points could be investigated in future studies.

## 4. The Metabolic Pathway of 2-AG Synthesis

The term was coined by Baggelaar et al. [13], referring to the discussion by Stella et al. [11] showing 2-AG as the main EC involved in modulating long-term potentiation. Current knowledge on lipolysis indicates that TAG hydrolysis occurs on the surface of intracellular lipid droplets essentially through the action of two lipases, i.e., PNPLA_2,_ also called ATGL (adipose triglyceride lipase), and DDHD2 [99,100,101,126,127,128]. Both are indicated in Figure 2, knowing that PNPLA_2_ predominates in adipose tissue but also displays a very broad expression profile, including the brain, whereas DDHD2 is specific to nervous tissue [99,100,128]. In the second step dealing with DAG hydrolysis, HSL appears as a possible candidate, as discussed in the previous paragraph. ABHD11 was not considered owing to its intramitochondrial localization (Figure 4). However, we also added DAGLβ, which was found to display a specific interaction with lipid droplets in Neuro-2a cells, in contrast to the typical positioning of DAGLα in the plasma membrane [46].

There is no experimental proof that the metabolic pathway is really involved in the generation of 2-AG. One argument against this hypothesis is the poor content of arachidonic acid in adipose tissue TAG (in the range of 0.3–0.5 mol percent, allowing the prediction of a maximum of 1% of TAG molecules being possibly converted into 2-AG) [129]. Also, in the brain of DDHD2^−/−^ mice, arachidonate-containing molecular species of accumulated TAG do not exceed 2% [65]. This casts some doubt on the real in vivo efficiency of the metabolic pathway in 2-AG synthesis. Indeed, studies dealing with adipocyte differentiation and the development of insulin resistance occurring in obesity related to overactive EC systems are focused on DAGLα rather than HSL [130,131]. A particularly attractive case might have been bone marrow adipocytes, which lack expression of MAG lipase, resulting in the accumulation of MAG [132]. However, 2-AG was not detected in that peculiar case. The same argument could be used for the synthesis of anandamide, whose first step involves the transfer of a fatty acid esterifying sn-1 position of PC, where AA is hardly present, to the amino group of PE (phosphatidylethanolamine), thus producing NAPE (N-acyl-PE) [133,134,135,136]. Although NAPE can then be converted to anandamide by multiple pathways, this step is currently considered as the unique obligate enzymatic reaction leading to anandamide synthesis.

A last argument against the metabolic pathway comes from the study comparing DAGL α and β in Neuro-2a cells differentiated by retinoic acid [46]. In that case, neurite outgrowth promoted by DAGLα is inhibited by a CB1 antagonist, according to the classical mechanism involving 2-AG, whereas the effect of DAGLβ, which is colocalized with lipid droplets, is independent of the 2-AG-CB1 axis. Although still hypothetical, we suggest that, in the latter case, TAG hydrolysis might contribute to other mechanisms involved in neurogenesis such as a balance between TAG and phospholipid biosynthesis [137].

## 5. The De Novo Synthetic Pathway

Turcotte et al. [138] reported a quantitative conversion of AA into 2-AG by human blood neutrophils in the presence of the serine esterase inhibitor MAFP (already mentioned in Section 2.4.4 [102]), i.e., under conditions affording the total inhibition of 2-AG hydrolysis. Other polyunsaturated fatty acids were also incorporated, with a maximum level for docosahexaenoic acid (DHA). Arachidonoyl-LPA (A-LPA) accumulation preceded that of 2-AG, suggesting the sequence of reactions described in Figure 2, i.e., the acylation of G3P followed by dephosphorylation by a phosphatase. Indeed, 2-AG production was almost abolished by inhibitors of arachidonoyl-CoA synthase and acyl-CoA transferase, triascin C, and thimerosal, respectively. The authors suggested the involvement of two acyltransferases, MBOAT5 and 7. The two enzymes actually correspond to lysophospholipid acyltransferases (LPLAT12 and LPLAT11, respectively, in a novel nomenclature [139,140]). These enzymes specifically acylate LPL, so that they do not appear as good candidates to synthesize 2-arachidonoyl-LPA. One attractive hypothesis would be the involvement of G3P acyltransferase2 (GPAT2), the mitochondrial enzyme displaying strong selectivity for AA, although its expression level in neutrophils is much lower than in spermatic cells [141,142].

Whereas the involvement of a lipid phosphatase could not be demonstrated using five different inhibitors, a very recent study provided some good evidence that the cytosolic enzyme PHOSPHO1, which dephosphorylates phosphocholine and phosphoethanolamine, would also be able to convert 2-arachidonoyl-LPA into 2-AG [143]. This is indicated in Figure 2. However, it still remains to be understood why 2-arachidonoyl-LPA would be converted into 2-AG rather than to AA-containing TAGs or PLs [142]. Indeed, in the de novo pathway of glycerolipid synthesis, LPA does not accumulate owing to the high activity of acylglycerol-3-phosphate acyltransferases (AGPATs). Confirming this fact, the pathological accumulation of LPA occurs in the liver and adipose tissue of rats upon the knockdown of Agpat2 (Lplat2 in the novel nomenclature [139,140]), thus affording an experimental model of the most common congenital lipodystrophy caused by inactivating mutations of the AGPAT2 gene (see Figure 2) [33].

Independently of the former study [138], GPAT2 was found to convert exogenous G3P into LPA in bone and bone marrow, revealing that both compounds behaved as extracellular messengers in a complementary way [144]. However, the latter study did not report a possible enrichment of LPA in AA nor the conversion of LPA into 2-AG. So, further investigations are required to clarify the possible role of the first step of glycerolipid de novo synthesis in the production of LPA and 2-AG. But, this remains an attractive hypothesis with possible pathophysiological relevance: (i) complementarity between kidney and bone in mineral metabolism [144]; (ii) the development of MAG hydrolysis inhibitors able to turn neutrophils into anti-inflammatory effectors through the production of 2-AG [138].

In a reverse way (see Figure 2), 2-AG and other MAGs can be phosphorylated into corresponding LPAs, as suggested in our former paper [76] and reviewed elsewhere [75,145]. Based on two studies characterizing purified proteins phosphorylating both diacyl- and 2-acyl-glycerols [146,147], Nakane et al. [148] also suggested the intracellular conversion of 2-AG into 2-A-LPA, which was currently included in reviews devoted to 2-AG synthesis [4,10,12,13,15]. In the light of the presently available knowledge, two types of enzymes can catalyze 2-AG phosphorylation: (i) among ten DAG kinases grouped in five different families (I to V), seven of them belonging to families I–III display significant activity (from 8 to 19% of DAG kinase activity) specifically towards 2-acyl-glycerol, with 1-acyl-sn-glycerol being a very poor substrate [149,150]; (ii) AGK was discovered as a multi-substrate lipid kinase (among which 2-AG) [151,152], is involved in cell proliferation and cancer [152,153], protein import into mitochondria [154], thrombopoiesis and thrombosis [155,156], and antitumor activity of CD8 T-cells [157], and is mutated in a rare recessive autosomal disease called Sengers syndrome [158]. It will thus be rather complex to decipher which of these enzymes might be involved in 2-AG phosphorylation in a given tissue. For instance, platelets, which have been the first example of an intact cell producing significant amounts of LPA [75,76,145], were found to express six different DAGKs [159] as well as AGK [156].

In this context, a recent study reported that the activation by orexin-A (OX-A, also called hypocretin 1 or HCRT1) of orexin receptor 1 (OX-1R) in cultured hypothalamic neurons induced the production of 2-A-LPA by a series of reactions involving the canonical pathway of 2-AG synthesis followed by the phosphorylation of the latter [160]. Tau phosphorylation was induced by both OX-A and 2-A-LPA in primary neuronal cultures and by 2-A-LPA in the hippocampal CA1 area upon intraperitoneal injection in a mechanism implying LPA1 receptor. Since OX-A, 2-AG, and 2-A-LPA concentrations were coordinately increased in plasma from Alzheimer patients, the authors pointed attention towards the possible involvement of that signaling pathway in relation to sleep disturbances occurring in Alzheimer disease [160,161], knowing the involvement of the orexin-orexin receptor system in the pathophysiology of narcolepsy type 1 [162]. A more recent study described the occurrence of the same pathway in hypothalamic neurons from arcuate nucleus, where decreased leptin signaling also led to 2-A-LPA synthesis, revealing its involvement in the regulation of appetite with obvious consequences on the development of obesity [163].

It thus appears that LPA and 2-AG metabolism can be closely related. This will also be obvious later when considering the AlterAG-2 pathway. However, as a main difference, enzymatic reactions described in Section 2, Section 3, Section 4 and Section 5 and in Figure 2 all display intracellular localization, whereas the last step of the three AlterAG pathways occurs on the cell surface and involves membrane ectoenzymes. In the first case, this implies that 2-AG has to cross the membrane to fulfill its function at the level of cannabinoid receptors. As discussed in several reviews [7,8,9,10], this can occur by simple diffusion but might also involve several proteins involved in intracellular transport (FABP5 or fatty acid-binding protein 5 [164,165]), transmembrane transport by still unidentified putative EMT (endocannabinoid membrane transporter [166,167]), or the release of EVs (extracellular vesicles [168,169,170,171]). As to the extracellular pathways presented in Figure 3, their detailed description in Section 7 will require the discussion of the importance of the sn-2 position of AA in 2-AG and its putative lysophospholipid precursors.

## 6. Importance of *sn*-2 Position of AA in 2-AG and LPLs

**Figure 5:**
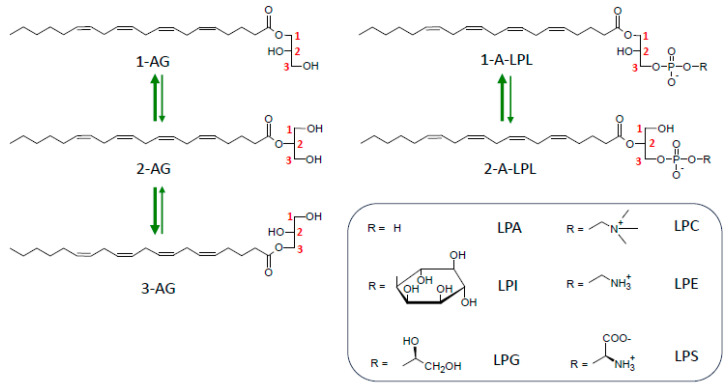
Acyl migration in monoacylglycerols and lysophospholipids. The most stable forms (1-AG and 3-AG, 1-A-LPL) represent roughly 90% of the mixture at the thermodynamic equilibrium. Abbreviations: 1-AG, 2-AG, 3-AG, 1 (or 2 or 3)-arachidonoylglycerol; 1-A-LPL or 2-A-LPL, 1 (or 2)-arachidonoyl-lysophospholipid; LPA, lysophosphatidic acid; LPC, lysophosphatidylcholine; LPE, lysophosphatidylethanolamine; LPG, lysophosphatidylglycerol; LPI, lysophosphatidylinositol; LPS, lysophosphatidylserine.

As shown in Figure 5, both MAG and LPLs undergo spontaneous migration of their unique acyl group to the vicinal hydroxyl group(s) of glycerol. Acyl migration leading to the formation of regioisomers was first reported by Emil Fischer, the famous chemist awarded the Nobel Prize in 1902 [172,173]. Since then, a large body of literature has been devoted to this problem (see, for instance, [174,175,176,177]). From these previous and from more recent studies [178,179,180,181], a consensual view has emerged: thermodynamic equilibrium corresponds to about 90% of 1(3)-AG or 1-acyl-LPLs versus 10% 2-AG or 2-acyl-LPLs; acyl migration occurs with first-order kinetics, is acid- or base-catalyzed, and has a maximal stability occurring at pH 4.0–5.0; 2-acyl compounds are more stable in a hydrophobic environment afforded by solvents or upon inclusion in membrane bilayers. In contrast, na aqueous medium accelerates migration, which is still more efficient in the presence of albumin. As a consequence of this, migration in LPLs was found to be the highest in serum compared to tissues [179]. This infers that even 1(3)-acyl glycero(phospho)lipids display some instability, since around 10% of them will spontaneously isomerize into their corresponding regional isomers under conditions used in functional assays, with most of them favorizing acyl migration (pH 7.4, presence of albumin). However, it is noteworthy that migration strongly decreases with the degree of unsaturation of the acyl chain [181].

These properties create a double issue: (i) one concerns the analytical tools necessary to determine the precise balance between regioisomers present in native biological media; (ii) the other one is to differentiate whether those isomers display regioselectivity towards specific enzymes and receptors. The two problems must be resolved to understand the biological relevance of a given metabolic pathway. Appropriate solutions to analytical problems are now available for LPLs and 2-AG as well [182,183,184,185], and we previously applied one of these methods [185] to successfully distinguish 2-AG from 1-AG [32]. On the other hand, precautions necessary to minimize acyl migrations have allowed us to draw conclusions about regioisomer reactivity towards various receptors and enzymes. The main available data are reported in Table 4.

### 6.1. Regioselectivity of Various Receptors

#### 6.1.1. CB1 Receptor

Despite some variations between studies, it seems to be generally agreed upon that CB1 displays regioselectivity towards 2-AG, with differences in potency of 2- and 1(3)-isomers varying between three- and ten-fold. This might reflect the use of different signaling events such as increases in cytoplasmic free [Ca^2+^] [186], [^35^S]GTPγS binding [187], or cAMP production [188]. In the latter study, Farah et al. reported EC_50_ of 96 nM, 480 nM, and 1450 nM for 2-AG, 3-AG, and 1-AG, respectively, indicating stereospecific recognition of the enantiomers 1- and 3-AG. A recent study [189] measuring the inhibition of EPSCs (excitatory postsynaptic currents) by 2- or 1(3)-AG in autaptic hippocampal neurons challenged the previous data of Stella et al. [11]. They concluded a total lack of activity of 1(3)-AG, whose apparent effects (at least 10-fold lower) might be attributed to the unavoidable 10% contamination by 2-AG occurring through acyl migration. However, one argument makes a case for a significant activity of 1(3)-AG. This concerns the comparison by Sugiura et al. [186] of the two ether-linked analogs of 2-AG and 1(3)-AG, where chain migration does not occur and which display the same relative difference in potency, albeit being both less powerful than their acyl counterparts. Finally, an intriguing but very carefully conducted study [190] reported that the mixture of 1(3)-AG and 2-AG formed within minutes upon acyl migration kept almost the same potency towards CB1-induced calcium mobilization as the initial 2-AG solution. Although three-fold less potent than 2-AG when tested alone, 1(3)-AG exerted with 2-AG additive effects which might be involved in situations of tonic CB1 receptor activation requiring prolonged incubations. In strong contrast, 1-AG was found completely inactive under conditions where 2-AG promoted cholecystokinin secretion from enteroendocrine cells [191]. However, this occurred in 60 min incubations at surprisingly high 2-AG concentrations (100 μM).

So, taking into account all these data is somewhat confusing and might lead to the idea that 2-AG acyl migration is not so important to consider in the context of its physiological function. However, this is only true if 2-AG is synthesized by the canonical pathway described above, where only the 2-isomer is initially produced. As we will discuss later, alternative pathways directly producing 1-AG would lead to a rather poorly active mediator displaying one order of magnitude lower potency. Since the three alternative pathways that will be discussed involve the conversion of LPLs to 2-AG, only 2-acyl-LPLs will be considered for our purposes.

#### 6.1.2. CB2 Receptor

At variance with CB1, we are aware of only one study comparing 2-AG and 1(3)-AG activity at CB2 (Table 4). The experimental approach conducted by Sugiura et al. [192] was essentially the same as that on CB1 [186], except that the neuronal cell line NG108–15 was replaced by HL-60 cells. Besides the demonstration of a regioselectivity of CB2 towards 2-AG isomers, this study clearly confirmed 2-AG as a full agonist of CB2, in contrast to anandamide. This reinforced the idea that 2-AG should play an important role in the immune system as well.

#### 6.1.3. TRPV1 Receptor

TRPV1 belongs to a large family of twenty-right transmembrane ion channels, six of them, including TRPV1, being activated by various cannabinoids. They are thus considered as ‘ionotropic endocannabinoid receptors’ [193]. By measuring Ca^2+^ influx in TRPV1-expressing HEK293 cells, Iwasaki et al. [194] reported the activation of TRPV1 by a number of MAG-bearing unsaturated fatty acids, including 2-AG and 1(3)-AG, which displayed identical activities. The latter conclusion on the lack of regioselectivity of 2-AG was confirmed by Zygmunt et al. [195]. So, ligand structural requirements and thus possible metabolic pathways leading to TRPV1 activation do not display the same strictness as in the case of metabotropic CB1 and CB2 receptors.

#### 6.1.4. GPR55 Receptor

An apparently broad specificity was also described for GPR55 towards various LPI species, including saturated and unsaturated long-chain fatty acids [197]. In fact, 2-arachidonoyl-LPI revealed to be around eight- to fifteen-fold more potent than other species and displayed a biological activity three times greater than that of 1-arachidonoyl-LPI, suggesting that 2-arachidonoyl-LPI might be the natural ligand of GPR55. However, that view was challenged by the observation that lysophosphatidyl-β-D-glucose (Lyso-PtdGlc), a novel lipid mediator involved in spinal cord sensory axon guidance, displayed an about seven-fold higher potency than LPI (respectively, EC_50_ 16 nM and 110 nM), inferring that LysoPtGlc rather than LPI would be the natural ligand of the recently deorphanized GPR55 receptor [198]. However, that comparison was carried out between 1-stearoyl species of both LysoPtdGlc and LPI, whereas the 2-arachidonoyl species of the latter would have been about 10-fold more potent [197]. By this time, the available information leaves open the possibility that both 2-arachidonoyl-LPI and LysoPtdGlc (and perhaps other saturated 1-acyl-LPI species) could be natural ligands of GPR55. Deciphering the peculiar enzymes responsible for their production might be the first step to allowing the precise description of their possible biological function in using appropriate models of knockout mice. This will be discussed further in the section on AlterAG pathways.

#### 6.1.5. LPA Receptors

Six different GPCR (LPA1–6) are now recognized as LPA receptors [199,200]. Although there is no strict rule in the definition of LPA species acting as ligands, two receptors display some preference. As recalled in Table 4, LPA3 (also called Edg7) and LPA6 (previous name, P2y5) both display a preference for 2-acyl-LPA, at a variance with the four other LPA receptors [201,203]. However, 2-arachidonoyl-LPA revealed to be at least 10-fold less potent than LPA bearing a Δ^9^ cis bond such as 2-oleoyl- or 2-linoleoyl-LPA [201,203]. Very surprisingly, the unnatural enantiomer of a phosphorothioate analog of LPA, (2S)-1-oleoyl-2-O-methyl-glycerophosphothioate ((2S)-OMPT), was found to be five- to twenty-fold more active than (2R)-OMPT [221]. Interestingly, the LPA3 tissue expression profile is very similar to that of membrane-associated PA-specific PLA_1_α (mPA-PLA_1_α), also known as lipase H (LIPH), which was found to couple 2-acyl-LPA production with LPA3 activation in an in vitro system [202]. Still, more obvious coupling between LIPH and LPA6 was found in hair follicles, where homozygous mutations of either LIPH or LPA6 result in a congenital hair deficiency called wooly hair/sparse hair or hypotrichosis (see [204] for a very elegant description of the pathophysiological mechanisms underlying that hair disorder and for references to discovered human mutations). Another involvement of LPA6 concerns differentiation into goblet cells of the colon carcinoma cell line HT-29, where LPA6 displays inhibitory effects, in strong opposition to LPA5 [205]. Differences in the reactivity of LPA6 and LPA5 were observed by using selective ligands (1-arachidonoyl-LPA and 1-*O*-alkyl-LPA for LPA6 and LPA5, respectively). However, 2-arachidonoyl-LPA would have been a more specific agonist of LPA6 [203]. Finally, the determination of the LPA6 structure combined to a docking simulation using 2-linoleoyl-LPA as a ligand provided interesting conclusions concerning the specificity of LPA6 ligand recognition as follows: whereas the phosphate group interacted with well-defined positive amino acid residues, the acyl chain was detected in a bent conformation within a cleft located between transmembrane domains TM4 and TM5 and was largely open to the lipid bilayer [206,207]. As discussed above, a direct transfer of 2-acyl-LPA to LPA6 within the lipid bilayer could maintain the stability of the 2-regioisomer, whereas 1-acyl-LPA would be prominent in the albumin-bound form present in the extracellular space, which is the preferential binding mode of LPA to LPA1 [222].

#### 6.1.6. LPS Receptors

Among the three LPS receptors identified in humans [208], LPS1 (also known as GPR34) was the only one displaying a strong preference for 2-acyl-LPS [208,209,210]. This was confirmed by comparing migration-resistant LPS analogs lacking *sn*-1 or *sn*-2 hydroxyl groups [209,210] and brought a strong argument for a possible functional coupling between LPS1 and PS-specific PLA_1_ (PS-PLA_1_) [223]. In a very recent and elegant study, LPS1’s tridimensional structure was resolved by cryo-electron microscopy with 1-oleoyl-LPS bound to the receptor, but did not provide any clue to explain the difference between *sn*-1 and *sn*-2 regioisomers [224].

### 6.2. Regioselectivity of Various Lipid Acyl Hydrolases

#### 6.2.1. MAGL, ABHD6, and ABHD12

Besides the well-characterized MAGL, two other hydrolases (ABHD6 and ABHD12) have been proposed as main enzymes regulating the 2-AG level in the brain [225,226]. Differences in cellular and subcellular localization might account for their complementary contributions to EC system homeostasis. As shown in Table 4, MAGL was found to be equally active against 2-AG and 1(3)-AG [211], which actually masked a stereospecificity revealed in a latter study as follows [188]: surprisingly, the three-fold higher V_max_ measured on 3-AG compared to 1-AG is coherent with the previous data. If we add the fact that the affinity of MAGL was the highest towards 1-AG [188], it remains difficult to relate those parameters to the well-established role of the enzyme in both adipose tissue lipolysis [99,100,101] and brain 2-AG homeostasis [225,226].

In contrast to MAGL, ABHD6 has a strong preference for 1- and 3-AG compared to 2-AG [188,211]. As previously reviewed [17,88], ABHD6 also displays high lysophospholipase activity against acidic LPLs such as LPG, BMP (also called LBPA), and possibly LPI [227,228,229,230]. Although only 1-acyl-LPLs were tested in the latter cases, one can reasonably extrapolate from MAG data that ABHD6 is less active on 2-acyl-LPLs.

The preference for external chains of MAG is also true for ABHD12 [211,212]. The latter enzyme, whose sequence predicts a luminal/extracellular localization, was first shown to catalyze extracellular 2-AG hydrolysis in microglia [84], in contrast to MAGL and ABHD6, which are active in the cytosolic compartment. However, the elucidation of the genetic defect responsible for the neurodegenerative disease PHARC (polyneuropathy, hearing loss, ataxia, retinosis pigmentosa, and cataract) revealed that ABHD12 deficiency actually resulted in the pathologic accumulation of LPS and, to a lower extent, of LPI [231,232]. The same in vivo accumulations can be partially reproduced using a specific inhibitor of ABHD12 [233,234]. Although the regional specificity of the enzyme was not examined on LPLs, the same preference for external chains can be inferred from data on MAG, as discussed above for ABHD6. Finally, the selective hydrolysis of long-chain LPS by ABHD12 seems to occur at an intracellular site, presumably the lumen of endoplasmic reticulum [212], which differs from the extracellular role played against microglial 2-AG, as recalled above [225].

#### 6.2.2. FAAH

FAAH is another enzyme able to hydrolyze 2-AG, although its natural substrates correspond to anandamide and its congeners *N*-acyl amides. As shown in Table 4, this occurs in the absence of any regioselectivity [188].

#### 6.2.3. PLRP2

PLRP2 was first described as a pancreatic lipase with high PLA_1_ activity [235] before being recognized as a member of the pancreatic lipase family, including a number of extracellular PLA_1_ [75,223,236,237,238]. The enzyme is actually expressed in a variety of tissues [239,240,241,242,243,244] and displays a rather broad substrate specificity, including neutral and phospho-glycerolipids, galactolipids [245], BMP [246], acylated PI-mannosides from phagocytosed mycobacteria [247], and retinyl esters [240]. However, as illustrated in Table 4, PLRP2 displays an exclusive specificity for the *sn*-1(3) positions, suggesting that it might be unable to hydrolyze acyl ester bonds involving a secondary alcohol, as previously discussed [214].

#### 6.2.4. LYPLA1 and LYPLA2

Although LYPLA1 and LYPLA2 are essentially lysophospholipases, they are also able to deacylate prostaglandin glycerol esters resulting from 2-AG oxygenation by cyclooxygenase 2 [215,216]. They also exert potent thioesterase activities catalyzing the depalmitoylation of heterotrimeric Gα subunits and Ras proteins [248]. As recalled in Table 4, the positional specificity of LYPLA2 towards arachidonoyl-glycerol and of both enzymes against LPLs is restricted to the *sn*-1(3) position under conditions minimizing acyl transfer.

#### 6.2.5. ABHD16A

ABHD16A was discovered in brain and macrophages as the major PS lipase producing the LPS substrate of ABHD12 discussed above [249,250,251]. Besides a specific role in LPS signaling, this activity, localized in endoplasmic reticulum [251], might be involved in mitochondrial fission and fusion events occurring at endoplasmic reticulum mitochondrial membrane contact sites [252]. In addition, similar to LYPLA1/LYPLA2, ABHD12 also displays depalmitoylase activity against Interferon-inducible transmembrane (IFITM) proteins [253]. In terms of regioselectivity, ABHD16A was described as a lipase directed against long-chain MAG (including 15-deoxy-prostaglandin J_2_ glycerol esters) with a clear preference for 1(3)-acyl regioisomers (Table 4, [217]). Whereas the latter finding might have led to predictions of PS-PLA_1_ activity, ABHD16A was found to deacylate PS at both the *sn*-1 and *sn*-2 position, as indicated by the fatty acid composition of LPS products [249]. To further add some mystery to that situation, the very close ABHD16B protein was described as a PS-specific PLA_1_ [254]. Whereas both isoforms contain a nucleophile motif essential to hydrolytic activity, like all but one ABHD proteins (ABHD15), only ABHD16A also contains an acyltransferase motif [255]. Whether such a structural difference has something to do with opposite regioselectivities remains presently unknown.

In conclusion, the examination of the regioselectivity of a number of lipid acyl hydrolases, although not exhaustive, still reveals a very complex world of enzymes exerting different enzymatic activities potentially corresponding to complementary functions. The situation is much simpler in the case of lipid phosphatases and phosphodiesterases.

### 6.3. Regioselectivity of Various Lipid Phosphatases and Phosphodiesterases

#### 6.3.1. LPPs

LPP1, LPP2, and LPP3 (gene names *PLPP1*, *PLPP2*, *PLPP3*) form a group (LPPs) of integral membrane proteins able to dephosphorylate PA, LPA, sphingosine 1-phosphate (S1P), ceramide 1-phosphate, and diacylglycerol pyrophosphate [256,257,258,259]. They belong to a larger family of lipid phosphatases/phosphotransferases comprising five different groups [256,257,258]. They will be discussed in more detail further with the description of the AlterAG-2 pathway. As far as we know, the possible regioselectivity of LPPs was never checked, but LPP1 was found to be non-stereospecific towards LPA itself [218] or a synthetic analog, *N*-acyl-norleucinol-1-phosphate [219] (Table 4). These observations are very coherent with the rather broad substrate specificity mentioned above; they suggest that LPP1 probably does not display a preference towards LPA regioisomers, which can be reasonably extended to LPP2 and LPP3.

#### 6.3.2. GDE3

As for GDE3 (*GDPD2* gene), its PLC activity is identical towards 1-acyl- and 2-acyl-LPI (Table 4), indicating a total lack of regioselectivity, thus allowing this enzyme to degrade all forms of LPI [32], as will be emphasized in the description of the AlterAG-1 pathway.

#### 6.3.3. ENPP6 and ENPP7

ENPP6 is also an ectoPLC acting on both 1-acyl- and 1-*O*-alkyl-LPC, platelet-activating factor sphingosylphosphorylcholine (SPC), *N*-acylethanolamine-*O*-phosphocholine, and glycerophosphocholine (GPC), which might be its natural substrate [260,261,262,263,264,265]. Two isomers, α-GPC (*sn*-glycero-3-phosphocholine) and β-GPC (*sn*-glycero-2-phosphocholine) are equally degraded [265]. The ability of ENPP6 to hydrolyze LPC as well as GPC is reminiscent of the activity of GDE3 on both LPI [31,32] and its deacylated product glycerophosphoinositol [266]. ENPP7 is another PLC with sphingomyelin as its main substrate (it is also named alkaline sphingomyelinase), together with LPC and PAF [260,261,267,268,269]. ENPP6 and 7 will be emphasized further as possible actors of the AlterAG-3 pathway upon acting on 2-arachidonoyl-LPC. Given the great variety of choline-containing substrates recognized by these enzymes, one can speculate that they lack regioselectivity, although experimental proof is not available.

#### 6.3.4. ATX

ATX (ENPP2) is the only secreted member of the ENPP family [260,261]. Following the discovery of its lysophospolipase D (lysoPLD) activity [270,271], it was universally recognized as the main enzyme involved in the last step of LPA production [260,261,272,273,274,275,276,277,278,279,280,281,282,283,284], although a specific pathway leading to 2-acyl-LPA also exists [273]. Besides LPC, which is the most abundant LPL in plasma, ATX can also hydrolyze LPE and LPS [285] as well as SPC [286], at least in vitro. Such a substrate specificity fits with the ATX tridimensional structure, which revealed a hydrophobic pocket, allowing the positioning of the acyl chain of lipid substrates in the vicinity of the catalytic site [287,288,289] (see also [274] for a very clear comprehensive review). As indicated in Table 4, one study revealed a relatively high level of regioselectivity of ATX [220]. This was observed by comparing ATX activity against 1-O-oleyl-*sn*-glycero-3-phosphocholine (lysoPAF-C18:1) and 2-O-oleyl-*sn*-glycero-3-phosphocholine, with these two lysoPAF regioisomers being protected from spontaneous interconversion by the stability of their O-alkyl bonds. This conclusion is in full agreement with the data obtained with sn-2-labeled LPL [290]. Bolen et al. [220] suggested that a main pathway of LPA production by activated platelets might involve the generation of 2-acyl-LPLs through the PLA_1_ activity of LYPLA1, followed by their spontaneous conversion into 1-acyl-LPLs, then allowing for the production of 1-acyl-LPA by ATX. As discussed further in Paragraph 7.2.5, this suggests that ATX might not be the most efficient way to generate 2-arachidonoyl-LPA as a precursor of 2-AG synthesis upon dephosphorylation by LPP. In addition to its hydrophobic pocket and active site, ATX also contains a partially hydrophobic tunnel able to bind LPA, thus acting as an LPA chaperone favorizing the delivery of the ligand to P2Y-type LPA receptors such as LPA6 [291]. There is no indication about a possible regioselectivity of LPA binding to the tunnel, rendering it difficult to understand the preference of ATX for the 1-acyl-LPC substrate and that of LPA6 for 2-acyl-LPA, as discussed above [203].

**Figure 6:**
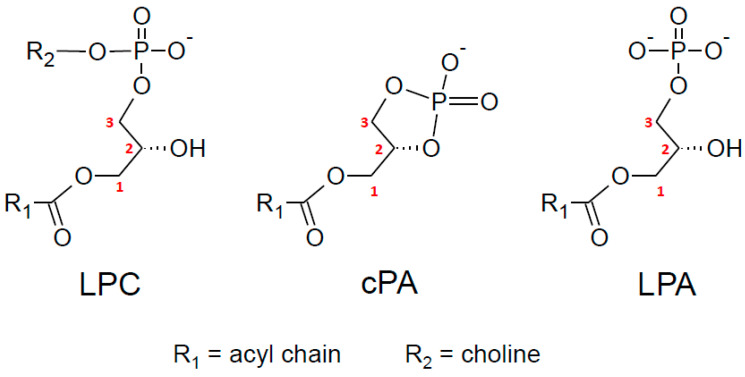
Structures of lysophosphatidylcholine (LPC), cyclic phosphatidic acid (cPA), and lysophosphatidic acid (LPA).

At this stage of the discussion, it is interesting to note that a lysoPLD from *Staphylococcus aureus* (*lpgD* gene) displays an absolute regioselectivity towards its 1-acyl-LPG substrate, in relation to the fact that cyclic phosphatidic acid (cPA), whose formation requires an *sn*-2-free hydroxyl group, appears in this case as an obligatory intermediate [292]. As very elegantly shown by the authors, the catalytic site is too small to accommodate at the same time that glycerol is released in the first step of the reaction where water is required to hydrolyze cPA. Like other lysoPLD, ATX was found to produce cPA involving the *sn*-2 hydroxyl group of the LPL substrate and is also able to hydrolyze cPA into LPA [293,294] (see the structures in Figure 6). As already suggested by Sano et al. [290], this might explain the regional preference of ATX for 1-acyl-LPL. However, there is no evidence that the ATX reaction mechanism involves a systematic cPA intermediate. The same question arises for other lysoPLD such as GDE4 and GDE7, which display strong differences in their ability to produce cPA [295,296]. But, there is no available indication yet for possible differences in the regioselectivity of GDE4 and GDE7.

In conclusion, it seems rather clear that the production and degradation of 2-AG requires specific attention being paid to the problem of regioselectivity. Its biological significance is difficult to draw in a straight way, given the number of receptors and enzymes concerned and described in the review. Still, the best example to illustrate possible biological significance is offered by ATX. The latter enzyme represents the major pathway of LPA production but displays an enzymatic activity almost restricted to 1-acyl-LPLs. In the case of LPA production by platelets in the context of thrombosis, this means that the production of LPA from 2-acyl-LPLs requires the delayed and progressive accumulation of 1-acyl-LPLs through acyl chain migration [220]. In addition, owing to its central role in LPA production, especially in the fields of lung sclerosis and tumor progression, a number of ATX inhibitors acting through various mechanisms have been developed, with an obvious advantage to tunnel-binding inhibitors [291]. It would be interesting to explore whether the ability of ATX to behave as an LPA chaperone involves regioisomerism or not. If this were not the case, such a dissociation might contribute to the different efficacy of inhibitors targeting the active site compared to tunnel-directed compounds.

A common feature of the three possible alternative pathways of 2-AG synthesis described in the following part of this review will concern a final step converting an LPL into 2-AG. This implies that only 2-arachidonoyl-LPL should be concerned.

## 7. AlterAG Pathways

### 7.1. AlterAG-1

#### 7.1.1. In Vitro Identification of GDE3 and DDHD1 as Main Actors of AlterAG-1

Thirty years ago, Ueda et al. [297] reported the presence of a PI-specific phospholipase A_1_ and a PLC degrading LPI into MAG in rat brains. After the identification of 2-AG as a major EC [11,298,299] two years later, a number of reviews on the EC system presented the sequence of a PLA_1_ and a lysoPLC as an alternative pathway producing 2-AG from PI [4,12,13,15,16,18]. In previous and forthcoming studies, LPI-specific PLC was characterized in various cells and tissues, including porcine platelets [300], fibroblasts [301,302], glioma cells and astrocytes [301], the brain, and synaptic membranes [303,304]. LPI-specific PLC was described as an ectoenzyme whose expression level was dramatically increased upon growth arrest [301,302].

**Figure 7:**
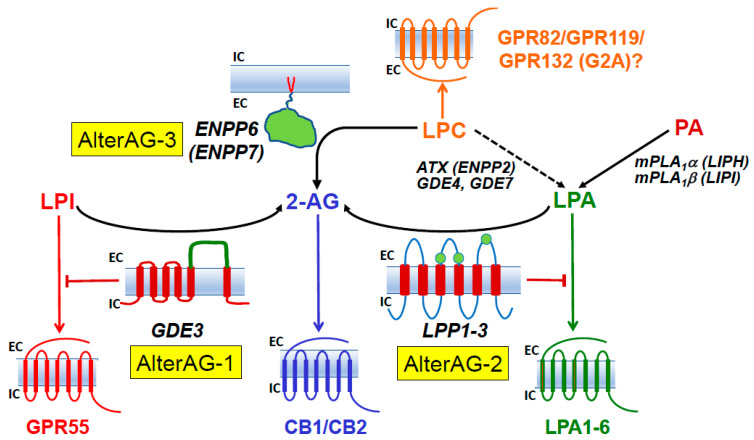
Extracellular reactions of AlterAG pathways. In AlterAG-1, GDE3 exposes to the cell exterior its catalytic site (green line), allowing the conversion of extracellular LPI into 2-AG, thus acting as a switch between GPR55 and CB1 or CB2 receptors. In AlterAG-2, LPPs also display an extracellular catalytic site (green circles), allowing a similar signaling switch between LPA and cannabinoid receptors. Among possible sources of LPA in AlterAG-2, the implication of mPLA_1_ (LIPH and LIPI) is considered the most probable compared to lysoPLD, owing to the preference of ATX for 1-acyl regioisomers [220] and the intracellular localization of GDE4 and GDE7, although GDE7 has also been described as an ectoenzyme [345]. In AlterAG-3, ENPP6 is anchored to the membrane by a glycosyl-PI anchor and allows the conversion of LPC into 2-AG. As not indicated here, ENPP6 is actually a homodimer with the two 55 kDa subunits being joined by a disulfide bridge [346]. The same reaction can also be catalyzed by ENPP7, which displays a single-pass transmembrane segment with a short cytosolic C-terminal end. Moreover, both ENPP6 and ENPP7 can generate soluble forms upon C-terminal partial proteolysis [262,264,347]. The signaling switch could occur between LPC receptors GPR82, GPR119, or GPR132 (G2A) and CB1/CB2, but with a number of restrictions discussed in the text. All the lysophospholipids described in this Figure are considered as 2-arachidonoyl species. For the sake of clarity, the various pathways leading to their formation are not indicated, except for LPA. Extracellular (EC) and intracellular (IC) faces of the plasma membrane are indicated. Abbreviations: 2-AG, 2-arachidonoylglycerol; AlterAG, alternative pathway of 2-AG synthesis; ATX, autotaxin; CB, cannabinoid receptor; ENPP, ecto-nucleotide pyrophosphatase/phosphodiesterase; GPR, G-protein-coupled receptor; LPA, lysophosphatidic acid; LPC, lysophosphatidylcholine; LPI, lysophosphatidylinositol; PA, phosphatidic acid; GDE, glycerophosphodiesterase; LIPH and LIPI, lipases H and I; LPP, lipid phosphate phosphatase; mPA-PLA_1_ (membrane-associated PA-selective PLA_1_ (α or β); PI, phosphatidylinositol.

Glycerophosphodiesterases (GDEs) form a large family of highly conserved enzymes from bacteria to mammalia [305,306,307]. Whereas mammalian enzymes share with their bacterial counterparts the ability to hydrolyze glycerophosphodiesters, they display more diverse substrate specificity. For instance, three members, GDE1 (*GDE1* gene), GDE4 (*GDPD1* gene), and GDE7 (*GDPD3* gene), are also able to hydrolyze acylated forms of glycerophosphodiesters, therefore catalyzing peculiar steps of N-acylethanolamine synthesis, including anandamide [133,134,135,136,308]. GDE3 was first described as a phosphodiesterase specific for glycerophosphoinositol but, at variance with GDE1, which acts as a PLD-like enzyme releasing glycerophosphate and inositol, GDE3 displayed a PLC-like activity, thus liberating glycerol and inositol 1-phosphate [266]. This prompted us to check the possibility that GDE3 might be the LPI-specific PLC previously described. Using exactly the same methodology as that described previously (i.e., [^3^H]inositol-labeled LPI as a substrate) [301,302,303,304], we found that HEK293T cells transfected with cDNA coding for GDE3 acquired lysoPLC activity displaying the same properties as the enzyme described thirty years ago: i) GDE3 is expressed in the plasma membrane; ii) it acts as an ectoenzyme; iii) it displays an optimal pH of 7.4; and iv) it requires mM Ca^2+^ for full activity [32]. This behavior is very similar to that already described with [^3^H]glycerophosphoinositol, including the abolition of activity upon R230A mutation and the lack of production of cyclic inositol-1,2-phosphate, at a variance with classical PLC [32,266]. The activity of GDE3 is limited to monomeric substrates, corresponding to the physiological concentrations of LPI reported in the literature. As already mentioned (Table 4), GDE3 was equally active against 1-acyl- and 2-acyl-LPI, but remained inactive on other LPLs (LPC, LPE, LPG, and LPS). The ectoenzymatic activity of GDE3 is fully coherent with its predicted transmembrane arrangement which, at a slight variance with initial proposals [305,306,309], contains six hydrophobic domains and an extracellular glycerophosphodiesterase domain [32,310,311] (see scheme in Figure 7). This arrangement is shared by two other members of the GDE family (GDE2 and GDE6). However, we were unable to detect PLC activity of GDE2 [32], whereas, to the best of our knowledge, GDE6 was never checked for this.

During the preparation of our manuscript [32], Tsutsumi et al. [31], using both lipidomic analysis and fluorescent substrates, reached the same conclusion with transfected COS-7 cells expressing GDE3. They also detected PLC activity with fluorescent diacyl-PI bearing a short chain, enabling them to insert in the outer layer of the surface membrane, as previously used by Ting and Pagano [312,313].

On the other hand, DDHD1, which was first identified as a PA-preferring PLA_1_ [314], was found to produce 2-arachidonoyl-LPI under conditions where PA played the role of a specific activator [315]. In addition, the localization of DDHD1 close to the plasma membrane (precisely focal adhesions) is regulated by phosphorylation of the protein [316].

Based on these findings, successive actions of DDHD1 and GDE3 can be proposed as forming the AlterAG-1 pathway, as depicted in Figure 3. Following a previous proposal concerning 1-acyl-LPI [317,318], this would require the involvement of the ATP-binding cassette transporter ABCC1 to export 2-arachidonoyl-LPI from the cell interior, rendering it available to the ectoenzyme GDE3 (see also [319,320] for reviews). Another possibility might be the cleavage of PI by an extracellular PLA_1_ such as PLRP2, as we suggested previously [32]. Whereas the majority of PI and other phosphoinositides seem to be confined to the cell interior owing to membrane phospholipid asymmetry [75,321,322,323], there is growing evidence that phosphoinositides such as PI 3-monophosphate (PI3P), PIP_2_, or PI 3,4,5-trisphosphate (PIP_3_) can also be present in the external leaflet of plasma membranes [324,325,326,327]. In this context, a PLA_1_ specific for PI3P was recently identified in *Vibrio cholerae* [328].

A main advantage of the AlterAG-1 pathway that is shared with the canonical pathway described in great detail above is the rather high abundance of arachidonic acid occupying the *sn*-2 position of PI [20,21]. However, one argument in favor of AlterAG-1 relevance would be to demonstrate the activity of its two enzymes in vivo.

#### 7.1.2. Signaling Switch between GPR55 and Classical Cannabinoid Receptors

The substrate and product of GDE3 are the ligands of GPR55 and CB1 (or CB2), respectively. As predicted, we have shown that the expression of GDE3 together with GPR55 abolished the Ca^2+^ signal induced by LPI, whereas 2-acyl-LPI promoted the same inhibition of adenylate cyclase as that evoked by 2-AG in CHO cells expressing both GDE3 and CB2 [32]. We thus concluded that GDE3 should act as a switch between GPR55 and cannabinoid receptors, as illustrated in Figure 7. The same proposal was made by Tsutsumi et al. [31], who suggested a possible role of GDE3 in bone remodeling through the increased expression occurring during osteoblast differentiation [266,309]. However, in vivo evidence for these suggested functions is still lacking. Furthermore, this might add a level of complexity to the fact that CB1 or CB2 are able to interact with GPR55, mainly by forming heteromers [329,330,331,332,333,334,335,336,337,338,339].

#### 7.1.3. Evidence That GDE3 and DDHD1 Are Functional In Vivo

GDE3 displays its highest expression levels in the spleen, small intestine, skin, bone, and bone marrow [32,306,340,341]. In contrast to previous detections of LPI-specific PLC activity in the brain mentioned above [297,303,304], GDE3 expression is much weaker in mouse brains, resulting in PLC activity about 40-fold lower compared to the spleen [32]. In agreement with the latter result, spleens from GDE3-KO mice displayed a significant accumulation of various LPI species associated with a decrease in 2-AG compared to wild type animals, whereas no changes were observed in brains [32]. So, there is at least one example indicating that GDE3 is active in vivo, giving some strength to the hypothesis of GDE3 being involved in 2-AG synthesis through the AlterAG-1 pathway.

On the other hand, by comparing DDHD1^+/+^ and DDHD1^−/−^ mice, Cravatt’s group observed a significant decrease in the arachidonoyl-LPI level in the brain of mice lacking DDHD1 and identified PI (and to some extent PS) as the natural substrate of DDHD1 in the brain [342]. This conclusion is very important when considering that DDHD1 in vitro activity is actually directed against a number of anionic phospholipids, including PI, PA, PG, PS, and BMP, as reviewed by Yaginuma et al. [223]. Another striking but still unexplained finding of this study was the observation that a rather minor species of polyunsaturated PI (1-oleoyl-2-arachidonoyl-PI) appeared to be specifically degraded by DDHD1, whereas no significant change occurred in the level of the major molecular species 1-stearoyl-2-arachidonoyl-PI. Finally, concerning DDHD1 being present in the spleen, probably in macrophages, it is interesting to note that 1-oleoyl-2-arachidonoyl-PI was increased in the spleen of DDHD1-KO mice without any decrease in arachidonoyl-LPI, in strong contrast to the brain. It is tempting to speculate that the high abundance of GDE3 in the spleen could hide any LPI accumulation, at variance with a healthy brain. Despite some differences in the data, another study comparing DDHD1^+/−^ and DDHD1^−/−^ fully confirmed the in vivo activity of this protein as a PI-specific PLA_1_ [343]. A still more recent study identified a specific increase in 1-oleoyl-2-arachidonoyl-PI in the plasma or serum of mice or humans bearing deleterious mutations of DDHD1 [344].

#### 7.1.4. Possible (Patho)physiological Role(s) of GDE3 and DDHD1

Despite the fact that GDE3 is active in vivo, at least in one organ where it displays a high expression level, the deletion of the GDE3 gene has not yet revealed any functional defect characterizing a given phenotype of GDE3-KO mice.

In the case of DDHD1, deleterious mutations are responsible for a very rare recessive hereditary spastic paraplegia, SPG28. Only 13 patients have been diagnosed and reported in seven publications, which are perfectly summarized in the last study on the subject [348].

From three mouse models of DDHD1 gene deletion published so far [342,343,349], only one reported behavioral alteration similar to those of SPG28 [343]. Those appeared only in 24-month-old animals, well after the appearance of metabolic changes related to DDHD1 deficiency. However, it is unclear whether the EC system is involved in the pathophysiology of SPG28, which might rather imply structural and functional alterations of mitochondria [349,350,351,352]. DDHD1 could also negatively regulate neurite outgrowth via the regulation of endosome recycling [353]. Finally, a compound heterozygosity for two canonical splice mutations in the *DDHD1* gene was detected in a male individual with autism spectrum disease, however, without any obvious relation to a defect in the EC system [354].

On the other hand, there are preliminary data reporting DDHD1 expression changes in some colorectal or esophageal cancers [355,356,357,358]. Corresponding discussions emphasize possible relations to the pro-tumorigenic role of the LPI-GPR55 axis, which might also be involved in tumor infiltration by T lymphocytes [356]. However, there are yet no available data emphasizing a possible role of GDE3 and AlterAG-1 in cancer.

#### 7.1.5. Other Activities of GDE3

A cutting-edge discovery was the demonstration that GDE2 acted as a PLC on glycosyl-PI anchors, inducing the shedding of a number of ectocellular proteins from the membrane [359]. Despite some variations between authors, transfected cells co-expressing GDE2 and glycosyl-PI-anchored proteins allowed for the identification of several substrates, among which were reversion-inducing cysteine-rich protein with Kazal motifs (RECK) [359,360] and various glypicans, mainly GPC4 and GPC6 [359,361]. These substrates have been validated in vivo using *Gde2*^−/−^ mice [360,362], and their list has been either confirmed or even extended by proteomic data obtained from Alzheimer disease [360] and amyotrophic lateral sclerosis (ALS) studies [363]. By involving either cell autonomous or non-autonomous mechanisms, GDE2 was identified as an important factor in embryonic neuronal [359] and non-neuronal [364] development, neurodifferentiation [361], neurodegeneration [360,362,363,365], and oligodendrocyte development [366,367].

The PLC cleavage of glycosyl-PI has also been recognized for GDE3 [310,359,368] and GDE6 [359,368]. From a functional point of view, the GDE3-induced shedding of the urokinase-type plasminogen activator receptor (uPAR) [310] and of the ciliary neurotrophic factor receptor α (CNTFRα) [311] results in the decreased proliferation of breast cancer cells and oligodendrocyte precursor cells (OPC), respectively. On the other hand, GDE6, whose expression appeared to be restricted to testes [305,306,307,369], is also present in chicken neural tube and controls its development [370].

From a biochemical point of view, cleavage by GDE-PLCs occurs in cis, i.e., in the same plasma membrane expressing both enzyme and substrate. In this context, appropriate trafficking to the plasma membrane of the protein is of crucial importance for proper function, as shown for GDE2 [371]. In addition, GDEs display very subtle differences in substrate specificity. For instance, uPAR shedding is promoted by GDE3 but remains refractory to GDE2 [310]. Again, GDE2 is unable to cleave glycosyl-PI anchors of Tiki proteins, in contrast to GDE3 and GDE6 [368]. Using homology modeling comparisons, van Veen et al. [310] provided clear evidence for striking differences in the surface charge distribution of GDE2 and GDE3, which might explain substrate selectivity.

These very important findings could cast some doubts on the possible role of GDE3 in the AlterAG-1 pathway. In fact, there are other examples of enzymes displaying different activities towards various substrates. For instance, cytosolic lysophospholipases LYPLA1-LYPLA2 (Table 4) are also thioesterases catalyzing the depalmitoylation of heterotrimeric Gα subunits and Ras proteins [215,216,248], whereas good evidence for a role of LYPLA1 as an extracellular PLA_1_ involved in the production of LPA during blood coagulation has been provided [220]. In the same manner, ABHD6 was discovered as a MAGL, but was also proposed to act as a DAGL as a lysophospholipase against BMP and other anionic lipids and to control AMPAR receptor trafficking [17,88,227,228,229,230].

In the absence of any data concerning GDE6, LPI-PLC activity thus appears as a unique property of GDE3 compared to GDE2. Another unique property of GDE3 resides in its capacity to promote the release of a very well-defined class of extracellular vesicles (EVs) from astrocytes [372]. The EVs are enriched in annexin-1 and GDE3, and the mechanism of their production involves the interaction of the cytosolic N-terminal end of GDE3 with actin cytoskeleton via Wiskott–Aldrich syndrome protein family member 3 (WAVE3). Moreover, GDE3-specific EVs were found to regulate miniature excitatory postsynaptic current (mEPSCs) amplitudes through an inhibitory action on mGluR1/R5. Interestingly, the authors suggested that the effects of GDE3-EVs on postsynaptic membranes might imply either the lysoPI or the glycosyl-PI PLC activity of GDE3 [372].

To close this part devoted to AlterAG-1, there are thus sufficient arguments justifying the exploration of the possible occurrence of this pathway in physiology and pathophysiology. In vivo data on spleen lipidomics and functional studies on astrocytes should justify further investigations. However, other important sites of GDE3 production such as the small intestine, skin, bone, and bone marrow could also be worthy of study.

### 7.2. AlterAG-2

#### 7.2.1. Discovery of AlterAG-2 Pathway

The seminal study describing what we suggest to call AlterAG-2 was published in 2002 by Nakane et al. [148]. They provided evidence that 2-arachidonoyl-LPA (together with 1-arachidonoyl species, probably upon acyl migration) represented 5.4% of the total LPA in rat brains. Moreover, they reported the conversion of 2-arachidonoyl-LPA into 2-AG by rat brain homogenates via a putative phosphatase, whose characterization was just beginning. Like the PLA_1_-lysoPLC cascade described above under the name AlterAG-1, this pathway was regularly included in a number of reviews where the reverse reaction catalyzed by a MAG kinase was also mentioned [4,10,12,13,15]. However, the knowledge on lipid phosphatases available at that time [373] was not included in the field.

#### 7.2.2. Signaling Switch between LPA and Classical Cannabinoid Receptors

As illustrated in Figure 7, there is a great similarity between AlterAG-1 and -2 in the sense that the last step catalyzed by LPPs also leads to a switch between LPA and cannabinoid receptors, as already suggested by Nakane et al. [148]. In this case, too, consequences of this switch might reach an increased level of complexity when considering the possible formation of heteromers between the two types of receptors, as shown for CB2 and LPA5 [374].

#### 7.2.3. Properties of LPPs

**Figure 8:**
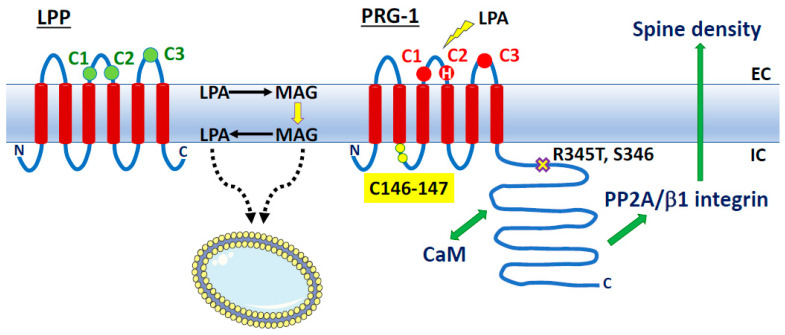
Comparative membrane insertion and functions of LPP and PRG-1. The three domains C1 to C3 forming the catalytic site of LPP (green) are colored in red to indicate the loss of phosphatase activity occurring in PRG-1 owing to several point mutations. H in domain C2 corresponds to His252, whose mutation abolishes interactions with LPA. R345T mutation produces a loss of function of PRG-1 by altering cytosolic O-glycosylation of neighboring S346 (as shown in a mouse model involving homologs R346T and S347A [428,440]. The lipid phosphatase activity of LPP converts LPA into MAG in the outer half of the plasma membrane. This is followed by the spontaneous translocation of MAG to the inner layer, allowing its possible reconversion into LPA by MAG kinase activity and the diffusion of both lipids to other intracellular membranes. The large cytoplasmic domain of PRG-1 was found to interact with CaM [427] and PP2A phosphatase, resulting in the last case in β1-integrin activation and allowing to maintain spine density [429]. Extracellular (EC) and intracellular (IC) faces of the plasma membrane are indicated. Abbreviations: CaM, calmodulin; LPA, lysophosphatidic acid; LPP, lipid phosphate phosphatase; MAG, monoacylglycerol; PP2A, protein phosphatase 2A; PRG1, plasticity-related gene 1.

As already discussed in Section 6.3.1, LPPs are lipid phosphate phosphatases with broad substrate specificity. As recently summarized in one of the most recent reviews on the subject [259], their structural arrangement involves six transmembrane segments and a catalytic site formed by three conserved domains (C1 to C3) present in second and third extracellular loops, also numbered III and V (Figure 7 and Figure 8). LPPs are present both in the plasma membrane, where they behave as ectoenzymes dephosphorylating the lipid mediators LPA and S1P, and in the endoplasmic reticulum and Golgi network, where their catalytic site faces the luminal side, with the PA being the presumed intracellular substrate [259,375].

Evidence for ectophosphatase activity involving mainly LPP1 and limiting LPA-induced biological responses was obtained in blood platelets [376,377] at a time where LPA5 was not yet recognized as a main LPA receptor present in those cells [378]. However, the inhibition of LPP1 by the mM calcium concentration present in the extracellular medium still remains somewhat mysterious when extrapolating to in vivo conditions [379]. In fibroblasts, LPP1 reduced LPA-induced cell migration by a dual mechanism involving not only ectophosphatase activity but also the intracellular hydrolysis of PLD-generated PA [380,381]. In endothelial cells, LPP3 was found to be specifically located at cell–cell contact sites, thus restricting the stress fiber formation evoked upon LPA6 activation to non-contact sites [382]. This provides a mechanism protecting endothelium from the alteration of its barrier properties caused by circulating LPA.

In vivo evidence for the ectophosphatase activity of LPP1 has been provided by measuring the increased plasma LPA concentration in hypomorphic mice displaying a low expression of LPP1 in most organs except the brain [383]. Interestingly, for our purpose, arachidonoyl-LPA occupied the second rank order among various LPA species detected in plasma from KO mice. Global postnatal decreases in LPP3 using conditional knockout under the control of an Mx1 promoter also resulted in increased plasma LPA [384]. On the other hand, hepatic LPP3 seems to play a key role in the turnover of plasma LPA, as revealed in mice with the conditional knockout of liver LPP3 [385]. In that case too, oleoyl-LPA and arachidonoyl-LPA were the most abundant species. Finally, the specific inactivation of cardiac LPP3 resulted in a three-fold increase in circulating LPA [386]. In contrast, the overexpression of LPP1 in transgenic mice did not result in a significant decrease in LPA plasma concentration [387], indicating that the mechanisms regulating LPA homeostasis might be more complex than anticipated. Moreover, a lack of gross modifications arising at the level of the whole animal might remain compatible with more subtle alterations occurring at very specific sites.

#### 7.2.4. (Patho)physiological Roles of LPPs

Despite some differences in the level of individual expression, LPPs are present in most tissues; however, they do not display redundance from a functional point of view. For instance, LPP1 and LPP2 knockout do not alter mouse viability [383,388], whereas LPP3 knockout induces embryonic lethality due to a defect in extraembryonic vasculogenesis [389]. Moreover, in a model of polarized MDCK cells, LPP1 and LPP3 are selectively addressed to apical and basolateral membrane, respectively, via specific motifs identified in their sequence [390]. They are also detected in different lipid rafts [391]. Finally, there is compelling evidence that LPP1 and LPP3, which are decreased in a number of tumors, have a protective role against tumor development and metastasis, probably by acting on both extracellular (mainly LPA) and intracellular substrates (for instance PA) (reviewed in [259]). In contrast, LPP2 might function as a tumor promoter, for still incompletely understood reasons [259,392]. As another difference, a recent study on breast cancer revealed that LPP1 and LPP3, whose expression is decreased, are mainly present in the tumor microenvironment (endothelial cells and cancer-associated fibroblasts), whereas LPP2 was concentrated and overexpressed in tumor cells [393].

On the other hand, in line with its role in embryonic vasculogenesis, LPP3 seems to play a key role in cardiovascular and metabolic diseases [394,395,396,397]. This is supported by several studies based on the conditional knockout of the *PLPP3* gene in various actors involved at various steps of atherosclerosis such as endothelial cells, myeloid cells (monocytes-macrophages), smooth muscle cells (SMCs), and cardiomyocytes [386,398,399,400]. One should add liver-specific knockout, resulting in an altered plasma lipid composition able to worsen atherogenesis in apoE^−/−^ mice [385]. These data are relevant to human pathology, with *PLPP3* being identified as a locus associated with coronary artery disease susceptibility, as reviewed in [394,395,396]. In addition, LPP3 expression is reduced in the human heart and adipose arterioles from patients with coronary artery disease [401]. As to possible mechanisms, one could recall the protective effect of LPP3 towards the endothelial barrier mentioned above [382]. A very interesting study reported that LPP3 displays a mechanosensitive expression under the control of microRNA-92a and transcription factor KLF2, with decreased expression occurring in response to disturbed flow and contributing to the altered local protection of endothelium through increases in available LPA [402]. In addition, LPA accumulated in atherosclerotic plaques was shown to trigger mastocyte-dependent plaque destabilization [403]. Surprisingly, in contrast to the atherogenic effect of LPA discussed above, its increase subsequent to LPP3-specific deletion in SMCs protects from angiotensin II-induced abdominal aortic aneurysm formation, probably by inducing the dedifferentiation of SMC into a fibroblast-like phenotype [404]. It is also interesting to note that the local and systemic application of 1-arachidonoyl-LPA promotes atherosclerosis in a mechanism involving LPA1 and LPA3, whereas stearoyl-LPA remains inactive in the same model [405]. Whether this could be replied with 2-arachidonoyl-LPA is presently unknown, but we will now discuss how 2-arachidonoyl-LPA could be produced in the context of the AlterAG-2 pathway.

#### 7.2.5. PLA_1_ (LIPH and LIPI) as a Major Pathway of 2-Arachidonoyl-LPA Production

As already discussed in paragraph 6.3.4., while ATX is broadly considered as the main pathway leading to LPA production, it might not be the privileged route to produce 2-arachidonoyl-LPA owing to its rather high selectivity for 1-acyl LPL species. This might also be true for other lysoPLDs such as GDE4 and GDE7, so the lysoPLD pathway is represented with a dotted line in Figure 7.

In contrast, another pathway based on PA-specific PLA_1_ has also been proposed [273] and regularly included in other reviews on the EC system [4,12,13,15]. That pathway was firmly recognized following the discovery of two specific PLA_1_, mPLA_1_α and mPLA_1_β, also called LIPH and LIPI [202,406,407,408]. Both enzymes belong to the pancreatic lipase gene family; they are membrane-associated and display an extracellular localization [223,237]. Both proteins show an affinity for heparin, suggesting that their membrane attachment might involve binding to heparan sulfate glycans, possibly glypicans [406]. The higher affinity of LIPI compared to LIPH seems to be related to the presence in the former of a region with higher contents of basic amino acid, similar to that found in other heparin-binding lipases such as endothelial lipase or lipoprotein lipase [409]. Interestingly, this interaction with glypicans might explain their specific localization in membrane microdomains (lipid rafts) [406], which has also been described for LPPs [391,410,411]. The concentration of LIPH/LIPI and LPP1/LPP2 in the same membrane microdomains, which also contain PLD2 [410], might allow strong coupling between the two enzymes of the AlterAG-2 pathway and their PA precursor. However, as discussed further, another major source of PA as a substrate of LIPH and LIPI might derive from phosphoinositides via PLC and DAG kinase.

LIPI shows a very selective localization in testes, where it is bound to the “connecting piece” of sperm, suggesting a specific function in male reproduction [406]. In line with this, LIPI was identified as a cancer/testes antigen (CTA), i.e., a group of immunogenic proteins showing predominant expression in gametogenic tissues and cancer and considered as interesting targets for anti-cancer vaccines [412,413]. It shows an almost exclusive overexpression in Ewing sarcomas [414]. Surprisingly, a deletion of *Lipi* exon 10 (*ldl* locus) in mice induces hepatic steatosis accompanied by hypertriglyceridemia, in keeping with a significant but low hepatic expression level [415]. The same study also reported several human mutations associated with altered plasma triglyceride or HDL cholesterol levels, suggesting “that mPA-PLA1b or its enzymatic products have a role in the metabolism in neutral lipids”, as commented by Aoki et al. [237]. A role of LPA in those metabolic regulations can thus be reasonably suggested, in agreement with data of hepatic LPP3 conditional knockout [385].

LIPH shows a much broader expression profile with the highest levels in the prostate, testes, ovary, colon, pancreas, kidney, lung, and platelets, but lower levels in the spleen, brain, and heart [202]. It is overexpressed in a number of cancers with a poor prognosis, probably related to the increased LPA production (see, for instance, [416,417,418,419]). As discussed in Paragraph 6.1.5 and reviewed in [273], an obvious function of LIPH is the specific activation of the LPA6 receptor by 2-acyl-LPA in hair follicles, as revealed by the homozygous inactivating mutations of either LIPH or LPA3 occurring in recessive wooly hair/hypotrichosis [204]. This can be reproduced in mice upon the knockout of *Liph* gene [420].

LPA production by activated platelets involves numerous enzymes including, among others, LYPLA1, ATX, and LIPH [220,285,290] (see also [75,421] for reviews). Instead of the previously suggested sPLA_2_ [422,423], the pathway leading to the successive formation of DAG, PA, and LPA through the actions of PIP_2_-specific PLC, DAG kinase, and LIPH thus appears as a privileged route ending with 2-arachidonoyl-LPA. It thus seems logical to consider the possibility of 2-AG production by LPP1 present at the surface of platelets [376,377]. Although further studies are required to quantitatively evaluate the contribution of LIPH (or LIPI) to 2-arachidonoyl-LPA synthesis, this pathway is indicated with a full line in Figure 7.

#### 7.2.6. The Enigmatic and Fascinating Case of LPR-4/PRG-1

In addition to LPP1–3, the integral membrane lipid phosphatase/phosphotransferase family includes a subfamily of five LPP-related proteins (LPR1 to 5, names of gene LPPR1 to 5), which are also called PRGs (plasticity-related genes) [256,257,424,425]. To limit our purpose to the field of the present review, we will focus on one isoform (PRG-1 or LPR-4), which is specifically located in the postsynaptic compartment (“postsynaptic density”) of glutamatergic excitatory synapses from both the hippocampus [426,427] and cortex [428].

As shown in Figure 8, three main differences with LPPs can be outlined as follows: i) the presence in PRG-1 of a 400–430 amino acid cytosolic C-terminal end able to bind calmodulin (CaM) [427] and PP2A protein phosphatase [429], promoting, in the last case, an LPA-induced increase in spine density as well as the modulation of synaptic plasticity and spatial memory; ii) two vicinal cysteines (C146–147), whose palmitoylation evoked by chemical long term potentiation appears to increase the insertion of AMPARs in the postsynaptic membrane [430]; and iii) the conservation of the three domains C1 to C3 forming the catalytic site, still with at least four point mutations of amino acid residues previously shown to be absolutely required for LPP1 phosphatase activity [431].

In contrast to previous conclusions [432,433], there is now a general consensus to admit that PRG-1 is devoid of lipid phosphatase activity [256,257,424,425,426,428]. Figure 8 recalls the mechanism by which LPPs promote the transmembrane transfer of LPA. This process was previously dissected using short-chain fluorescent PA which is able to intercalate in the outer monolayer of the cell membrane [434,435,436], but we infer that the same sequence of events might apply to LPA itself, since the spontaneous transmembrane movement of MAG is well established from the example of 2-AG (see various discussions above). However, the inactive phosphatase PRG-1 is also required for the same transfer to occur, as shown using either short-chain fluorescent PA [426] or fluorescent LPA [428]. Translocation activity disappears in neurons from PRG-1^−/−^ mice [426] or in transfected HEK293 cells upon the mutation of His252 [428], which is thought to be necessary for PA or LPA recognition by PRG-1. Interestingly, LPA internalization is also accompanied by the intracellular appearance of its metabolite MAG, in a way very similar to the mechanism involving LPP depicted in Figure 8. There is no definite explanation allowing the understanding of the ability of PRG-1 to internalize LPA, which might involve PRG-1-catalyzed translocation, PRG-1-driven endocytosis, or complementarity with an endogenous LPP. The latter hypothesis could be borrowed from the example of myotubularin 3-phosphatases, whose activity on PI3P and PI(3,5)P_2_ can be regulated by association with “dead enzymes” [437,438], as also described for other pseudoenzymes [439].

**Figure 9:**
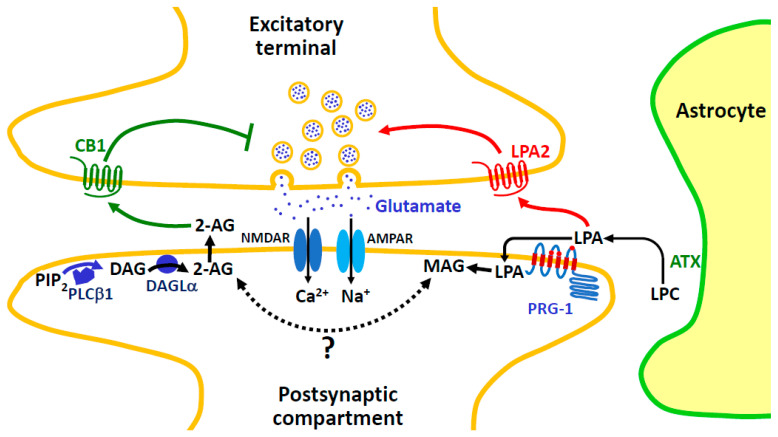
Regulation of excitation/inhibition balance at glutamatergic synapses by opposite effects of LPA and 2-AG. Glutamate released from synaptic vesicles activates postsynaptic neurons by interacting mainly with its ionotropic receptors AMPAR and NMDAR, which results in the depolarization of postsynaptic neurons, allowing Ca^2+^ influx through NMDAR (as well as voltage-gated Ca^2+^ channels, not represented here). In the left part of this Figure, the canonical pathway of 2-AG synthesis (as described in Figure 1) allows 2-AG to reduce glutamate secretion upon interaction with presynaptic CB1. In addition, 2-AG can also increase excitability by suppressing inhibitory GABAergic signals (not represented here). In the right part of this Figure, LPA produced through LPC hydrolysis by ATX secreted from astrocyte induces hyperexcitability upon binding to presynaptic LPA2 receptor. However, this effect is counteracted by the removal of LPA from the synaptic cleft by postsynaptic PRG-1. Thus, both DAGLα (via CB1) and PRG-1 (upon removing pro-excitatory LPA) contribute to reduce hyperexcitability at glutamatergic synapses. The same localization of DAGLα and PRG-1 in postsynaptic membrane generates the question of a possible mixing of 2-AG and of MAG produced as the consequence of LPA internalization by PRG-1 (curved dotted double arrow). Abbreviations: 2-AG, 2-arachidonoylglycerol; AMPAR, α-amino-3-hydroxy-5-methyl-4-isoxazole-propionic acid receptor; ATX, autotaxin; CB1, cannabinoid receptor 1; DAG, diacylglycerol; DAGLα, DAG lipase α; LPA, lysophosphatidic acid; LPA2, LPA receptor 2; LPC, lysophosphatidylcholine; NMDAR, N-methyl-D-aspartate receptor; PIP_2_, phosphatidylinositol 4,5-bisphosphate; PLCβ1, phospholipase C-β1; PRG1, plasticity-related gene 1.

Whatever the precise mechanism, the PRG-1-dependent uptake of LPA contributes to reducing LPA concentration in the synaptic cleft. As summarized in Figure 9 and as supported by a series of studies [426,428,441,442,443,444,445,446], astrocytes stimulated by glutamate via ionotropic receptors release ATX, resulting in the accumulation of LPA, which increases the probability of glutamate secretion via presynaptic LPA2 receptor. By removing the excess of LPA, PRG-1 thus reduces or even suppresses hyperexcitability without altering basal glutamatergic activity. Within the numerous studies cited above, experimental models and translational investigations demonstrate the main interest of this regulation in several pathological states, including psychiatric disorders [428,442,443,444], epilepsy [447], aging [445], stroke-induced damages [440], or even hyperphagia [446]. They also open interesting perspectives about the possible use of ATX or LPA2 inhibitors in those various pathologies.

In this context, a single nucleotide polymorphism (SNP) present as a monoallelic form in 0.6% of population (representing about 5 million people among European and US citizens) introduces a point mutation (R345T) inducing a loss of function of PRG-1 (see Figure 8). The electrophysiological exploration of two transgenic models (heterozygous deletion in PRG-1^+/−^ mice [426] or PRG-1^R346T^ mice reproducing the human SNP [428]) as well as human PRG-1^R345T^ mutation carriers [428,444] provided strong evidence that SNP significantly altered cortical excitation/inhibition balance.

To come back to 2-AG synthesis, there is an apparent link between PRG-1 and the EC system, since 2-AG produced by the canonical pathway also contributes to modulate glutamatergic hyperexcitability (Figure 9). However, a main difference between the two systems is the absence of LPA2 in GABAergic terminals, where CB1 is also expressed, giving rise to DSI, in addition to DSE (see Section 1 and Section 2).

Another question raised by the comparison of the two pathways concerns the possible relationship between MAG accumulated in the postsynaptic membrane and 2-AG produced by the canonical pathway. Both PRG-1 [426] and DAGLα [26,27,28,29] are present at the postsynaptic density of glutamatergic synapses, in interaction or in close proximity with the scaffold proteins Homer. As already discussed, ATX might not be the best enzyme to generate 2-arachidonoyl-LPA and indeed oleoyl-LPA rather than arachidonoyl-LPA accumulates in cerebrospinal fluid (CSF) under conditions (overnight fasting) inducing an ATX-sensitive increase in cortical excitability [446]. However, the question still remains as to whether PRG-1-derived MAG and 2-AG are mixing in the same leaflet of the postsynaptic membrane and why (and how) 2-AG is able to diffuse through the synaptic cleft to reach CB1, as already discussed at the end of Section 5.

As can also be discussed for the EC system, the role of PRG-1 might not be restricted to excitatory neurons, since it is also expressed in gastric cancer, promoting peritoneal metastasis [448] and in vascular smooth muscle cells, where it inhibits LPA-induced cell migration and proliferation [449]. The discussion of the latter paper is particularly interesting in relation to the role of unsaturated LPA species, including arachidonoyl-LPA, in atherosclerosis.

Finally, it is our duty to mention that, using either hypoglossal motoneurons [450], hippocampal CA1 pyramidal neurons [160], or orexin-A-expressing neurons from lateral hypothalamus [163], two other teams reported that LPA can act as an inhibitory retrograde messenger at presynaptic terminals in a mechanism involving LPA1 instead of LPA2 receptor. Together with the fact that an LPA1-driven inhibition also seems to be present in GABAergic terminal [450], these data are contradictory to those summarized in Figure 9. However, no comments about this obvious discrepancy are found in the corresponding papers, including a review devoted to glutamatergic transmission [451]. Further studies are thus needed to explain such differences.

### 7.3. AlterAG-3

A third extracellular pathway able to synthesize 2-AG might be the direct conversion of 2-arachidonoyl-LPC by PLC. On a theoretical basis, two enzymes, ENPP6 and ENPP7, could achieve this task, as illustrated in Figure 7. As already mentioned in Paragraph 6.3.3., both proteins are ectoenzymes, with ENPP6 possessing a glycosyl-PI anchor, whereas ENPP7 displays a single membrane pass with a short cytosolic C-terminal end [260,261]. We speculated that they lack regioselectivity, with LPC remaining a common substrate within a rather broad list of choline-containing phospholipids. However, in contrast to AlterAG-1 and -2, there is yet neither in vitro nor in vivo evidence that ENNP6 or 7 could contribute to 2-AG synthesis.

#### 7.3.1. Availability of Arachidonoyl-LPC as Substrate of ENPP6 and ENPP7

LPC occurs in plasma from normal human controls in the range of 150–300 μM, with 5–8 μM arachidonoyl-LPC [452,453,454,455]. The majority of plasma LPC is secreted by the liver, with arachidonoyl-LPC being the most abundant [456,457], but 2-acyl-LPC can also be generated in the intravascular compartment by the PLA_1_ cleavage of PC by enzymes such as hepatic lipase and endothelial cell-derived lipase (LIPC and G, respectively) [223,237,458], or LYPLA1 [220]. The production of 2-arachidonoyl-LPC can occur by additional mechanisms in other sites, for instance, upon the chemical degradation of choline plasmalogens by hypochlorous acid generated by myeloperoxidase in atherosclerotic lesions [459]. Among other biological fluids, CSF contains much lower concentrations of LPC [460]. However, LPC is the molecule engaged in the efficient transport of polyunsaturated fatty acids through the blood–brain barrier via Mfsd2a (major facilitator superfamily domain-containing protein 2A) [458,461,462,463,464]. LPC, together with other LPL, is also present in significant amounts in peritoneal fluid, as illustrated, for instance, in a model of carcinomatous peritonitis [465].

#### 7.3.2. Present Status of GPCRs Recognizing LPC as Specific Ligand

As for AlterAG-1 and -2, a signaling switch between LPL and cannabinoid receptors could be suggested, as indicated in Figure 7. However, the situation is not so clear for LPC receptors belonging to the class of GPCRs. For instance, GPR82 was described as an apparently constitutively active Gi-coupled receptor recognizing LPC as well as LPE as inverse agonists. Edelfosine (1-O-octadecyl-2-O-methyl-sn-GPC) was significantly more potent than 1-oleoyl-LPC [466], but nothing is known about the possible activity of 2-arachidonoyl-LPC on GPR82.

The best argument for GPR119 as an LPC receptor was the identification of endogenous LPC in the GPR119-Gs complex observed by cryo-EM [467]. Confirming or preceding several reports [468,469,470], LPC was found to activate adenylate cyclase [467], resulting in a potentiation of insulin release by pancreatic β-cells [468,469,470]. On the other hand, a much higher efficiency was obtained with APD668, a clinical drug candidate for type 2 diabetes [467]. In addition, 2-arachidonoyl-LPC was not identified among the molecular LPC species spontaneously bound to GPR119 [467]. However, as shown by others, GPR118 can bind various other lipidic ligands such as 2-oleoyl-glycerol, N-oleoyl-ethanolamine, or N-oleoyl-dopamine ([471] and references herein), putting the accent again on oleoyl rather than arachidonoyl side chain.

The situation is somewhat more confusing with GPR132 (also called G2A), which, together with GPR4, was initially recognized as an LPC receptor in two papers that were later retracted. As reviewed in [472,473], GPR132 and GPR4 actually belong to a group of proton-sensing receptors, with GPR132 regulation involving rather lactate molecules, with interesting implications in the interaction of macrophages with tumor [474] or apoptotic cells [475]. However, there is accumulated evidence that LPC exerts various biological effects in cells expressing GPR4 [476] or GPR132 [477,478,479,480,481], including T cells, macrophages, neutrophils, and endothelial cells. In line with these in vitro data, GPR132 gene invalidation increases susceptibility to late-onset autoimmunity or to atherosclerosis [482,483], whereas LPC displays protective effects against sepsis [484,485]. One possible mechanism of LPC interaction with G2A might involve an indirect effect on the membrane [480] and (or) the redistribution of the receptor by modulating its intracellular trafficking [480,486]. Adding more complexity to the problem, oxidized derivatives of linoleic and arachidonic acid [487], various N-acylamides [488], or commendamide (3-hydroxy-N-palmitoyl glycine, produced by human microbiome [489]) are also able to activate GPR132, with a role in hematopoiesis [490] and type 2 diabetes [491]. There is still an abundance of literature on G2A/GP132, whose exhaustive description would be out of the scope of the present review.

Finally, both agonistic and antagonistic activities of LPC towards Toll-like receptors have been described [492] but are not indicated in Figure 7.

#### 7.3.3. Properties of ENPP6 and ENPP7

The tissue expression of ENPP6 is the highest in the brain, cerebellum, kidney, testes, prostate, and ovary [493]. Morita et al. [265] provided good evidence for GPC being its natural substrate, allowing oligodendrocytes to acquire the choline necessary for myelin biosynthesis. On the other hand, the same ectoenzyme present in epithelial cells from kidney proximal tubules might allow choline reabsorption from primary urine. Although poorly expressed in the liver, ENPP6 is still present on the surface of sinusoidal endothelial cells, allowing choline uptake by hepatocytes. In line with this, Enpp6 gene invalidation induced fatty liver and demyelination, which are hallmarks of choline deficiency [265]. In this context, ENPP6 expression in newly forming oligodendrocytes was found to play a critical role in motor skill learning [494], whereas genetic or proteomic data revealed some interesting links between ENPP6 and Alzheimer disease [495,496].

ENPP6 is also present in matrix vesicles participating in mineralization. There is good in vitro and in vivo evidence that a cascade involving PLA_2_, ENPP6, and PHOSPHO1 would generate the inorganic phosphates necessary for mineralization [497,498]. However, it remains to be understood how ectoenzyme ENPP6 and cytosolic phosphatase PHOSPHO1 (see the preceding discussion in Section 5) might act in concert. This probably involves specific transporters.

As to ENPP7, it is the only enzyme from the ENPP family able to hydrolyze sphingomyelin and was discovered as alkaline sphingomyelinase [260,261,267,268,269]. It is located in the apical membrane of enterocytes [499], from which it can be solubilized upon partial cleavage by trypsin [347]. Solubilized enzymes acquire higher in vitro activity, suggesting that they might be involved in sphingomyelin digestion, with a membrane-bound enzyme generating ceramide from mucosal sphingomyelin [347]. However, both forms are probably involved in sphingomyelin digestion, as clearly demonstrated with ENPP7-KO mice [500]. The same mouse model allowed the demonstration of a protective role of ENPP7 against colonic tumorigenesis [499] and dextran sulfate sodium-induced colitis [501], the latter study confirming previous data in rats [502]. As reviewed by Duan [503], three mechanisms might be involved in the increased susceptibility of ENPP7-KO mice to cancer and inflammation as follows: decreases in apoptotic ceramide accumulation, increases in proinflammatory PAF, another substrate of ENPP7 [269], and a lack of LPC degradation into monoacylglycerol [268], leading to the stimulated production of LPA by ATX. Although not emphasized in previous studies, the possible production of 2-AG from 2-arachidonoyl-LPC by ENPP7 could also be considered, the latter substrate being potentially produced upon the hydrolysis of dietary PC by PLRP2 [75,214,235,236,237,238]. As already mentioned in Paragraph 7.1.3, GDE3 is highly expressed in enterocytes from the small intestine [32,305,340,341], although its presence in either apical or basolateral membrane has not yet been defined. This point would deserve further investigation in order to check whether the two lysoPLCs (ENPP7 and GDE3) do play, or not, complementary functions in intestinal 2-AG synthesis.

ENPP7 is also present in the human liver (see [503] for review), and we briefly mention recent studies describing phenotypic modifications occurring in ENPP7 KO mice, including intestinal and liver transcriptome [504,505], as well as the homeostasis of intestinal T lymphocyte populations [506]. Finally, possible links between the ENPP7 gene and type 2 diabetes [507] or gastric cancer [508] were recently reported.

To obtain AlterAG-3, in vivo evidence of its involvement in 2-AG synthesis is still lacking. The last paragraph below will pay attention to the possible use of ENPP6 and ENPP7 KO mice, which revealed interesting phenotypes. Lipidomic analysis would thus be worth performing in order to check the possible involvement of the EC system in the observed functional modifications.

## 8. Concluding Remarks and Potential Future Research Directions

This review thus provides an enormous amount of information, largely wider than the simple problem of 2-AG synthesis. By linking endocannabinoid to LPL metabolism and signaling, it extends the already rather complex world of LPL mediators [509]. Obviously, most of the proposals concerning AlterAG pathways are still hypothetical and require further investigations, taking advantage of the existence of specific KO mice models such as those dealing with GDE3, ENPP6, or ENPP7. This has been already performed with LPPs, but conclusions were mainly, if not exclusively, focused on the possible consequences of LPA accumulation. In other words, any further investigation should consider the possibility of a double effect of GDE3, ENPP6, ENPP7, or LPP suppression. We can reasonably predict that, depending on the case and on the tissue, either increased LPL levels or 2-AG suppression, or both, might be responsible for the observed functional changes. Any clarification should involve either pharmacological probes or the use of double-KO mice models (for instance GDE3 and CB1 (or CB2) double KO, as well as GDE3 and GPR55 double KO).

In addition to the use of mouse transgenic models, which should clarify the potential pathophysiological functions of AlterAG pathways, this field could take great advantage of modern imaging methods able to localize 2-AG and LPLs in tissues, for instance, by mass spectrometry imaging [24,403] or spatiotemporally resolved in vivo imaging using genetically engineered fluorescent sensors [22,23] (see also [5] for review).

Another striking observation stemming from this survey concerns possible functional redundancy between LPL and 2-AG signaling. This is, for instance, the case depicted in Figure 9, where 2-AG synthesis and LPA removal will both contribute to regulating excitation–inhibition balance at glutamate synapses in a process where the two metabolic pathways are or not related.

Redundancy was also noted between different activities displayed by the same protein; this is the case for ABHD6 (MAG but also DAG lipase and lysophospholipase) and LYPLA1 (lysophospholipase, thioesterase, and PLA1). This renders conclusions difficult to draw, especially when using inhibitors [510], but can correspond to multiple functions of those enzymes. It is also the case of GDE3 when considering PLC activity towards LPI and glycosyl-PI anchors.

It is tempting and reasonable to explore functional consequences of GDE3, ENPP6, or ENPP7 knockout in those tissues displaying the highest expression level of the corresponding enzyme. However, opposite situations might also exist. For instance, GDE3 is hardly expressed in the lung, while the role of GDE3 present in club cells was revealed in a model of allergic airway inflammation [511]. Among many possibilities of interesting studies to develop, we reasonably can expect that the field covered by AlterAG pathways will provide a number of interesting discoveries in the coming years. One can speculate that some of these discoveries might reveal the involvement of AlterAG pathways in the pathophysiology of various diseases already known to imply endocannabinoids and (or) lysophospholipids, from psychiatric disorders to inflammation, cancer, or cardiovascular diseases. As an illustration of these possibilities, a study was published just after the submission of our review, showing that soluble epoxide hydrolase (sEH, gene name Ephx2, or Abhd20), a cytosolic bifunctional enzyme bearing N-terminal lipid phosphate phosphatase and C-terminal epoxide hydrolase activities [512,513,514], was involved in the balance between LPA and 2-AG, specifically in the olfactory bulb [515]. This was shown by the selective genetic deletion of the lipid phosphate phosphatase activity of sEH, resulting in a depressive-like behavior of mice bearing the mutation [515]. The same approach in the rat revealed that sEH knock-in prevented obesity and cardiac ischemia–reperfusion injury [516]. Interestingly, the EPHX2 gene was also recently identified by Mendelian randomization as a therapeutic target for Alzheimer disease [517]. These unexpected data are a good example of what can be reached with functional studies on AlterAG pathways.

The latter finding is so important that we included sEH in Figure 2 as the most probable enzyme achieving the intracellular conversion of 2-arachidonoyl-LPA to 2-AG. That pathway is somewhat different from AlterAG-2, which displays an extracellular localization, but the relationship between the two pathways will deserve peculiar attention in the near future.

## Figures and Tables

**Table 2:** Main characteristics of PLC isoenzymes possibly involved in 2-AG synthesis.

PLC Subtype	Activation Mechanism	Main Localization Tested	References
PLCβ1	Gα_q_	Hippocampus	[25]
PLCβ4	Gα_q_	Cerebellum	[35]
PLCδ1,δ2,δ3	Ca^2+^ (10 μM)	Brain	[36,70,72,73]
PLCη1,η2	Ca^2+^ (1 μM)	Brain	[70,71]
PLCε	cAMP via Epac	Ventral tegmental area	[37]
PLCγ1	Tyr phosphorylation	Brain	[38,39]
PLCγ2	Tyr phosphorylation	Myeloid cells	[40]

Abbreviations: Gα_q_, heterotrimeric G-protein with α_q_ subunit; Epac, exchange protein directly activated by cAMP; PLC, phospholipase C.

**Table 3:** Effect of tetrahydrolipstatin (THL) on various serine hydrolases.

Serine Hydrolases	Substrate	Effect of THL	References
DAGLα	DAG	Inhibited	[41]
DAGLβ	DAG	Inhibited	[41]
ABHD12	MAG, LysoPS	Inhibited	[103]
ABHD16A	PS	Inhibited	[103]
TPP2	Tripeptide	Inhibited	[103]
PLA2G7	PAF	Inhibited	[103]
HSL	DAG > TAG > MAG	No effect	[103]
DDHD2	DAG > TAG	Inhibited	[63]

Abbreviations: ABHD, α/β-Hydrolase Domain-Containing; DAG, diacylglycerol; DAGL, DAG lipase; DDHD, DDHD containing; HSL, hormone-sensitive lipase; MAG, monoacylglycerol; PAF, platelet-activating factor or 1-*O*-alkyl-2-acetyl-*sn*-glycero-3-phosphocholine; PLA2G7, group 7 phospholipase A_2_; PS, phosphatidylserine; TAG, triacylglycerol; TPP2, tripeptidyl peptidase 2.

**Table 4:** Compared biological properties of 2-AG and LPL regioisomers.

Receptors	Ligand or Substrate Preference	References
CB1	2-AG > 3-AG > 1-AG	[11,186,187,188,189,190,191]
CB2	2-AG > 1-AG = 3-AG	[192]
TRPV1	2-AG = 1(3)-AG	[193,194,195]
GPR55	2-A-LPI most potent among LPI species	[196,197,198]
LPA3	2-acyl-LPA > 1-acyl-LPA (2-arachidonoyl-LPA < 2-oleoyl-LPA)	[199,200,201,202]
LPA6	2-acyl-LPA > 1-acyl-LPA (2-arachidonoyl-LPA < 2-oleoyl-LPA)	[199,200,203,204,205,206,207]
GPR34	2-acyl-LPS > 1-acyl-LPS	[208,209,210]
**Enzymes**		
MAGL	3-AG > 2-AG > 1-AG; 1(3)-AG = 2-AG	[188,211]
ABHD6	1-AG = 3-AG > 2-AG	[188,211]
ABHD12	1(3)-AG > 2-AG	[211,212]
FAAH	1-AG = 2-AG = 3-AG	[188]
PLRP2	1(3)-acyl-*sn*-glycerol > 2-acyl-glycerol = 0;1-acyl-LPC >> 2-acyl-LPC = 0	[213,214]
LYPLA1/LYPLA2	1(3)-AG >> 2-AG = 0;1-palmitoyl-LPC >> 2-palmitoyl-LPC = 0	[215,216]
ABHD16A	1(3)-linoleoyl-*sn*-glycerol > 2-linoleoyl-glycerol	[217]
LPP1–3	LPP1 non stereospecific	[218,219]
GDE3	2-acyl-LPI = 1-acyl-LPI	[32]
ENPP6–7	Not determined	
ATX/ENPP2	1-O-alkyl-LPC >> 2-O-alkyl-LPC	[220]

Abbreviations: ABHD, α/β-Hydrolase Domain-Containing; AG, arachidonoylglycerol; ATX, autotaxin; CB, cannabinoid receptor; ENPP, ecto-nucleotide pyrophosphatase/phosphodiesterase; FAAH, fatty acid amide hydrolase; GDE3, glycerophosphodiesterase 3; GPR, G-protein-coupled receptor; LPA, lysophosphatidic acid; LPA3 or LPA6, LPA receptors 3 or 6; LPC, lysophosphatidylcholine; LPI, lysophosphatidylinositol; LPP, lipid phosphate phosphatase (types 1 to 3); LPS, lysophosphatidylserine; LYPLA1 or 2, lysophospholipase A1 or 2; MAGL, monoacylglycerol lipase; PLRP2, pancreatic lipase-related protein 2; TRPV, transient receptor potential cation channel subfamily V.

## Abbreviations

2-AG, 2-arachidonoylglycerol	GABA, γ-amino butyric acid
AA, arachidonic acid	GDE, glycerophosphodiesterase (types 1 to 7)
ABHD, α/β-Hydrolase Domain-Containing	GPAT, sn-glycerol-3-phosphate acyltransferase
AGK, acylglycerol kinase	GPC, glypican
AGPAT, 1-acylglycerol-3-phosphate acyltransferase	GPCR, G-protein-coupled receptor
A-LPA, arachidonoyl-LPA	GPR55, G-protein-coupled receptor 55 (other types with different numbers)
AlterAG, alternative pathway of 2-AG synthesis	HSL, hormone-sensitive lipase
AMPAR, α-amino-3-hydroxy-5-methyl-4-isoxazole-propionic acid receptor	I-LTD, long-term depression at inhibitory synapses
AP4, adaptator protein complex 4	LBPA, lysobisphosphatidic acid
ATGL, adipose triglyceride lipase	LIPH and LIPI, lipases H and I
ATX, autotaxin	LPA1 to 6, LPA receptors 1 to 6
BDNF, Brain-Derived Neurotrophic Factor	LPC, lysophosphatidylcholine
BMP, bis(monoacylglycero)phosphate (see also LBPA)	LPE, lysophosphatidylethanolamine
CaM, calmodulin	LPG, lysophosphatidylglycerol
CB1, cannabinoid receptor (types 1 or 2)	LPI, lysophosphatidylinositol
CNTFRα, ciliary neurotrophic factor receptor α	LPL, lysophospholipid
cPA, cyclic phosphatidic acid	LPLAT, lysophospholipid acyltransferase
D609, tricyclodecan-9-yl-xanthogenate	LPP, lipid phosphate phosphatase (types 1 to 3)
DAG, diacylglycerol	LPR1 to 5, LPP related proteins (see also PRG)
DAGK, diacylglycerol kinase	LPS, lysophosphatidylserine
DAGL, diacylglycerol lipase	LPS1 to 3, LPS receptors 1 to 3
DDHD, DDHD containing	LYPLA1 or 2, lysophospholipase A1 or 2
DHA, docosahexaenoic acid	Lyso-PtdGlc, lysophosphatidyl-β-D-glucose
DSE, depolarization-induced suppression of excitation	MAFP, methylarachidonoylfluorophosphonate
DSI, depolarization-induced suppression of inhibition	MAG, monoacylglycerol
EC, endocannabinoid	MAGL, monoacylglycerol lipase
ENPP, ecto-nucleotide pyrophosphatase/phosphodiesterase	mGluR1/5, metabotropic glutamate receptor (1 or 5)
Epac, exchange protein directly activated by cAMP	mPA-PLA_1_ (membrane-associated PA-selective PLA_1_ (α or β)
EVs, extracellular vesicles	MPSIIIB, mucopolysaccharosidosis type IIIB
FAAH, fatty acid amide hydrolase	NAGLU, N-acetyl-β-D-glucosaminidase gene
G3P, sn-glycerol-3-phosphate	NAPE, N-acyl-phosphatidylethanolamine
NMDAR, N-methyl-D-aspartate receptor	PNPLA_2_, patatin-like PLA_2_
NODRS, neuro-ocular DAGLA-related syndrome	PP2A, protein phosphatase 2A
OGDHc, oxoglutarate dehydrogenase complex	PPAR, peroxisome proliferator-activated receptor
OX-A, orexin-A; OX-1R, orexin receptor 1	PRG1 to 5, plasticity-related genes
PA, phosphatidic acid	PS, phosphatidylserine
PAF, platelet-activating factor or 1-O-alkyl-2-acetyl-sn-glycero-3-phosphocholine	RECK, reversion-inducing cysteine-rich protein with Kazal motifs
PC, phosphatidylcholine	S. nigra, substantia nigra
PE, phosphatidylethanolamine	sAC, soluble adenylate cyclase
PG, phosphatidylglycerol	sEH, soluble epoxide hydrolase
PHOSPHO1, phosphocholine and phosphoethanolamine phosphatase	SMS, sphingomyelin synthase
PI, phosphatidylinositol	SMSr, sphingomyelin synthase-related protein
PI3P, phosphatidylinositol 3-monophosphate	TAG, triacylglycerol
PIP_2_, phosphatidylinositol 4,5-bisphosphate	TAGL, triacylglycerol lipase
PIP_3_, phosphatidylinositol 3,4,5-trisphosphate	THC, Δ-9-tetrahydocannabinol
PKA or PKC, protein kinase A or C	THL, tetrahydrolipstatin (lipase inhibitor)
PLA_2_, phospholipase A_2_	TPA, 12-O-tetradecanoylphorbol-13-acetate
PLC, phospholipase C	TRPV, transient receptor potential cation channel subfamily V (various types with different numbers)
PLD, phospholipase D	uPAR, urokinase-type plasminogen activator receptor
PLRP2, pancreatic lipase related protein 2	VTA, ventral tegmental area
